# Supplementary material for: Simultaneous determination of free and total metabolite concentrations in proteinaceous specimens by 1D 1H CPMG NMR
Source: Cell Rep Methods. 2026 Jan 16;6(1):101291. doi: 10.1016/j.crmeth.2025.101291 (PMC12853166; doi:10.1016/j.crmeth.2025.101291)
Supplement: Document S1. Figures S1–S4 and Tables S1–S4 [file mmc1.pdf]

Cell Reports Methods, Volume *6*

Supplemental information

Simultaneous determination of free and total metabolite concentrations in proteinaceous specimens by 1D ^{1}H CPMG NMR

Alexander Reindl, Claudia Samol, Silke Haerteis, Helena U. Zacharias, Katja Dettmer, Peter J. Oefner, and Wolfram Gronwald

Table S1. NMR characteristics of model compounds and their optimal linewidths L_{oM} in the absence of protein (29 metabolites and TSP). Related to STAR Methods subheading "IMPLEMENTATION OF THE CORRECTION ALGORITHM".

Note that for multiplets with highly overlapping signals the width of the whole multiplet measured at half height will be used. All linewidths were experimentally determined in pure water at 298 K.

metabolite	chemical shift and atoms[1]	signal characteristic	optimal linewidth L_{oM} [Hz]
3-Hydroxyisovalerate	2.4 ppm, (**H**2)$_2$	singlet	1.39
3-Indoxyl sulfate	7.7 ppm, (**H**4)	full multiplet	10.42
Acetate	1.9 ppm, (**H**2)$_3$	singlet	0.74
Acetoacetate	2.3 ppm, (**H**4)$_3$	singlet	1.47
Acetone	2.2 ppm, (**H**1)$_3$, (**H**3)$_3$	singlet	0.73
Alanine	1.5 ppm, (**H**3)$_3$	left peak of doublet	1.03
Citrate	2.7 ppm, (**H**2B)	left peak of doublet	1.25
Creatine	3.0 ppm, (**H**5)$_3$	singlet	0.96
Creatinine	4.0 ppm, (**H**2)$_2$	singlet	0.96
Ethanol	1.2 ppm, (**H**2)$_3$	middle peak of triplet	0.75
Formate	8.4 ppm, (**H**1)	singlet	0.70
Glucose	3.5 ppm, (**H**3-b)	singlet	1.22
Glycine	3.6 ppm, (**H**2)$_2$	singlet	0.77
Hippurate	7.8 ppm, (**H**6, **H**10)	full multiplet	10.46
Hypoxanthine	8.2 ppm, (**H**8)	left peak	1.40
Isobutyrate	1.1 ppm, (**H**3)$_3$, (**H**4)$_3$	both peaks together	7.82
Isoleucine	1.0 ppm, (**H**6)$_3$	left peak of doublet	1.18
Lactate	1.3 ppm, (**H**3)$_3$	left peak of doublet	0.87
Leucine	1.0 ppm, (**H**5)$_3$, (**H**6)$_3$	middle peak of multiplet	1.76
Lysine	3.0 ppm, (**H**6)$_2$	middle peak of triplet	1.92
Methanol	3.4 ppm, (**H**1)$_3$	singlet	0.72
N,N-Dimethylglycine	2.9 ppm, (**H**4)$_3$, (**H**5)$_3$	singlet	0.87
Phenylalanine	7.3 ppm, (**H**5), (**H**9)	full multiplet	9.98
Pyruvate	2.4 ppm, (**H**3)$_3$	singlet	0.73
Succinate	2.4 ppm, (**H**2)$_2$, (**H**3)$_2$	singlet	0.80
Threonine	3.6 ppm, (**H**2)	right peak of doublet	0.89
Tryptophan	7.7 ppm, (**H**9)	full multiplet	10.38
TSP	0.0 ppm, (**H**5)$_3$, (**H**6)$_3$, (**H**7)$_3$	singlet	0.78
Tyrosine	6.9 ppm, (**H**6), (**H**8)	full multiplet	9.60
Valine	1.0 ppm, (**H**4)$_3$	left peak of doublet	1.11

[1]All atoms were named according to the nomenclature provided by the Bruker BBIOREFCODE database (Bruker BioSpin GmbH, Ettlingen, Germany).

Table S2. Validation of the linewidth-based correction factors on plasma pools. Related to Table 2.

The table compares the correction factors obtained by the new algorithm with those from protein precipitation. (A) Plasma pool no. 2. (B) Plasma pool no. 3. All concentrations were determined by NMR quantification. Column 7 shows the concentrations of the metabolites after ultrafiltration of the sample and column 8 relates those concentrations to the free metabolite concentrations. Here, for most metabolites the quotients of filtrated and free concentration scatter around 1.0. Note that Table S2A does not include threonine as it could not be adequately quantified in this spectrum. Instead, values for succinate are shown, which could not be sufficiently quantified in the spectra of Tables 2A and S2B.

(A) metabolite	free [mM] (2)	precipitated [mM] (3)	(3)/(2)=(4)	algorithm (5)	(4)/(5)	filtrated [mM] (7)	(7)/(2)
Acetate	0.166	0.193	1.165	1.071	1.088	0.171	1.033
Alanine	0.157	0.179	1.146	1.114	1.029	0.165	1.051
Citrate	0.134	0.131	0.975	1.001	0.974	0.123	0.915
Creatine	0.021	0.021	1.029	1.028	1.001	0.019	0.942
Creatinine	0.064	0.080	1.238	1.224	1.011	0.069	1.079
Glucose	6.615	6.941	1.049	1.052	0.997	6.276	0.949
Glycine	0.121	0.135	1.115	1.098	1.015	0.095	0.783
Isoleucine	0.043	0.047	1.112	1.034	1.075	0.039	0.920
Lactate	1.027	1.063	1.035	1.106	0.936	0.986	0.960
Leucine	0.086	0.098	1.143	1.123	1.017	0.079	0.920
Lysine	0.085	0.093	1.092	1.157	0.944	0.089	1.040
Phenylalanine	0.043	0.050	1.164	1.191	0.977	0.035	0.812
Pyruvate	0.072	0.082	1.132	1.140	0.993	0.089	1.234
Succinate	0.015	0.019	1.224	1.257	0.974	0.016	1.072
Tyrosine	0.037	0.045	1.203	1.146	1.050	0.041	1.102
Valine	0.174	0.200	1.155	1.079	1.071	0.175	1.008
(B) metabolite	free [mM] (2)	precipitated [mM] (3)	(3)/(2)=(4)	algorithm (5)	(4)/(5)	filtrated [mM] (7)	(7)/(2)
Acetate	0.070	0.086	1.225	1.214	1.009	0.075	1.064
Alanine	0.239	0.283	1.188	1.146	1.037	0.256	1.074
Citrate	0.151	0.161	1.069	1.029	1.039	0.140	0.929
Creatine	0.037	0.044	1.200	1.095	1.096	0.037	1.005
Creatinine	0.083	0.108	1.302	1.298	1.003	0.092	1.102
Glucose	6.918	7.381	1.067	1.092	0.977	6.344	0.917
Glycine	0.141	0.161	1.149	1.144	1.004	0.107	0.764
Isoleucine	0.058	0.068	1.164	1.210	0.962	0.067	1.146
Lactate	1.539	1.632	1.061	1.191	0.891	1.618	1.052
Leucine	0.112	0.139	1.242	1.191	1.043	0.113	1.008
Lysine	0.087	0.110	1.257	1.180	1.065	0.108	1.236
Phenylalanine	0.046	0.061	1.335	1.331	1.003	0.054	1.175
Pyruvate	0.092	0.110	1.202	1.197	1.005	0.119	1.301
Threonine	0.090	0.090	1.003	1.023	0.980	0.094	1.047
Tyrosine	0.052	0.073	1.404	1.316	1.067	0.062	1.191
Valine	0.208	0.244	1.172	1.106	1.060	0.218	1.049

Table S3. Comparison of the linewidth-based correction algorithm with LC-MS/MS and an IDMS-traceable enzymatic assay in five different plasma specimens. Related to Table 2.
Validation of the total concentrations of selected amino acids and creatinine determined by application of linewidth-based correction factors against the absolute concentrations measured either by an IDMS-traceable enzymatic assay (creatinine) or by stable-isotope dilution LC-MS/MS. (A) plasma specimen 1. (B) plasma specimen 2. (C) plasma specimen 3. (D) plasma specimen 4. (E) plasma specimen 5.

(A) metabolite	NMR, free conc. [mM] (2)	correction factor (3)	NMR, total conc. [mM] (4)	MS, total conc. [mM] (5)	(5/2)	(5/4)
Alanine	0.188	1.28	0.240	0.249	1.33	1.04
Creatinine	0.065	1.26	0.082	0.088	1.36	1.08
Glycine	0.204	1.12	0.228	0.237	1.16	1.04
Isoleucine	0.038	1.09	0.041	0.047	1.24	1.14
Threonine	0.111	1.05	0.117	0.116	1.04	0.99
Tyrosine	0.037	1.34	0.049	0.049	1.33	0.99
Valine	0.156	0.99	0.155	0.186	1.19	1.20
(B) metabolite	NMR, free conc. [mM] (2)	correction factor (3)	NMR, total conc. [mM] (4)	MS, total conc. [mM] (5)	(5/2)	(5/4)
Alanine	0.212	1.21	0.256	0.266	1.26	1.04
Creatinine	0.077	1.51	0.117	0.126	1.63	1.08
Glycine	0.113	1.32	0.149	0.167	1.48	1.12
Isoleucine	0.029	1.04	0.030	0.041	1.42	1.38
Threonine	0.085	1.04	0.088	0.087	1.03	0.99
Tyrosine	0.055	1.36	0.075	0.075	1.36	1.00
Valine	0.153	1.03	0.158	0.168	1.10	1.07
(C) metabolite	NMR, free conc. [mM] (2)	correction factor (3)	NMR, total conc. [mM] (4)	MS, total conc. [mM] (5)	(5/2)	(5/4)
Alanine	0.191	1.07	0.204	0.236	1.24	1.16
Creatinine	0.084	1.30	0.109	0.126	1.50	1.15
Glycine	0.141	1.21	0.171	0.194	1.38	1.14
Isoleucine	0.034	1.01	0.035	0.041	1.20	1.18
Threonine	0.067	0.92	0.062	0.074	1.10	1.19
Tyrosine	0.031	1.28	0.040	0.041	1.32	1.03
Valine	0.134	1.03	0.137	0.148	1.11	1.08
(D) metabolite	NMR, free conc. [mM] (2)	correction factor (3)	NMR, total conc. [mM] (4)	MS, total conc. [mM] (5)	(5/2)	(5/4)
Alanine	0.282	1.07	0.303	0.333	1.18	1.10
Creatinine	0.141	1.40	0.197	0.210	1.49	1.06
Glycine	0.092	1.13	0.104	0.129	1.40	1.25
Isoleucine	0.044	1.11	0.049	0.057	1.30	1.17
Threonine	0.070	1.02	0.071	0.075	1.08	1.05
Tyrosine	0.035	1.28	0.045	0.048	1.37	1.08
Valine	0.155	1.03	0.160	0.168	1.09	1.05
(E) metabolite	NMR, free conc. [mM] (2)	correction factor (3)	NMR, total conc. [mM] (4)	MS, total conc. [mM] (5)	(5/2)	(5/4)
Alanine	0.196	1.14	0.222	0.233	1.19	1.05
Creatinine	0.096	1.36	0.131	0.145	1.50	1.11
Glycine	0.146	1.15	0.168	0.164	1.13	0.98
Isoleucine	0.055	1.05	0.058	0.068	1.24	1.18
Threonine	0.075	1.22	0.091	0.079	1.06	0.87
Tyrosine	0.028	1.35	0.038	0.038	1.34	0.99
Valine	0.194	1.02	0.198	0.200	1.03	1.01

Table S4. Linewidth-based correction factors for six selected amino acids and creatinine in five different plasma specimens – summary. Related to Table 2.

Shown are the metabolite-specific linewidth-based correction factors obtained for each of the five GCKD plasma specimens (Table S3) and the mean (±SD) of the metabolite-specific correction factors obtained for the five specimens. The last column gives for each metabolite the average agreement in concentrations between MS and corrected NMR data as a ratio of the two.

metabolite	sample 1	sample 2	sample 3	sample 4	sample 5	mean±SD	mean MS vs mean corrected NMR data
Alanine	1.28	1.21	1.07	1.07	1.14	1.15±0.09	1.08
Creatinine	1.26	1.51	1.30	1.40	1.36	1.37±0.10	1.10
Glycine	1.12	1.32	1.21	1.13	1.15	1.19±0.08	1.11
Isoleucine	1.09	1.04	1.01	1.11	1.05	1.06±0.04	1.21
Threonine	1.05	1.04	0.92	1.02	1.22	1.05±0.11	1.02
Tyrosine	1.34	1.36	1.28	1.28	1.35	1.32±0.04	1.02
Valine	0.99	1.03	1.03	1.03	1.02	1.02±0.02	1.08

A

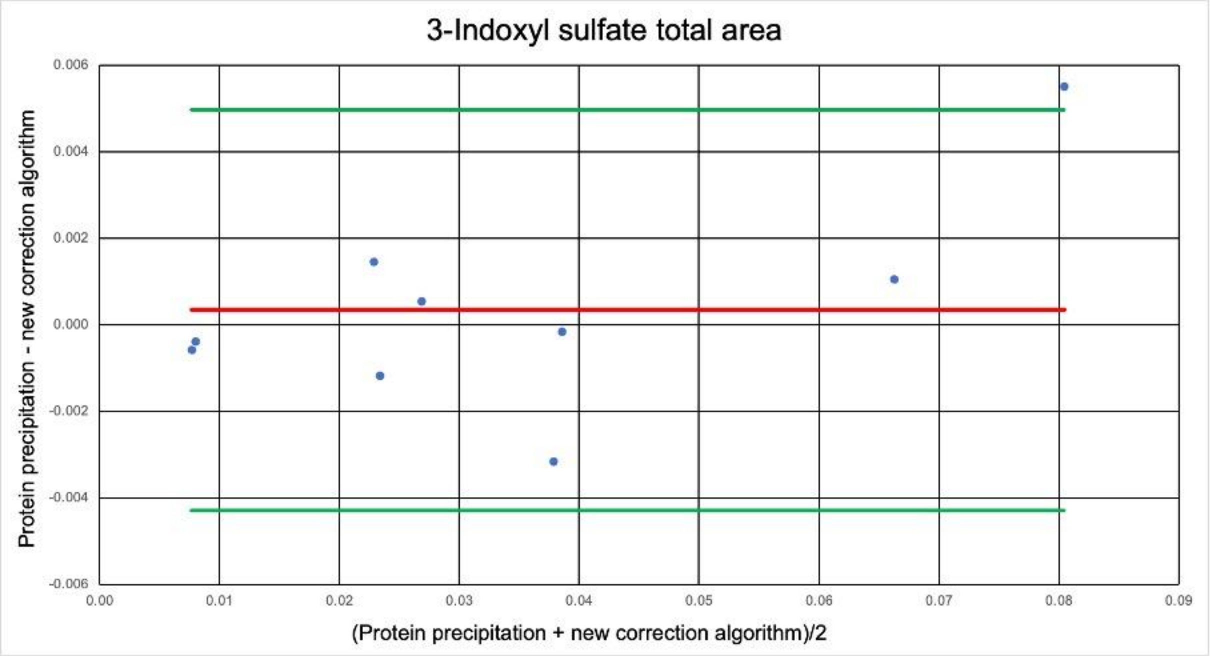

B

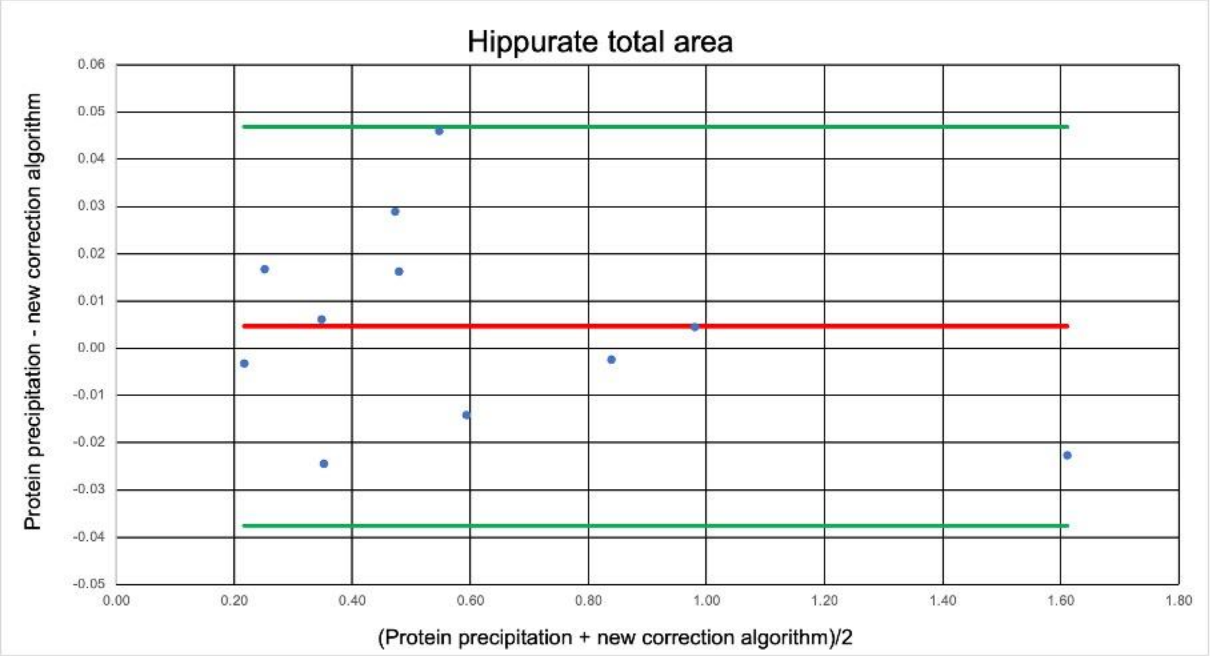

Figure S1. Comparison of the total area of 3-indoxyl sulfate (A) and hippurate (B) in urine specimens determined by the linewidth-based correction algorithm versus protein precipitation. Related to Figure 2.

The Bland-Altman plots show the differences between the peak integrals for total (A) 3-indoxyl sulfate and (B) hippurate determined by 1D ^{1}H CPMG NMR after protein precipitation of urine with methanol and the use of linewidth-based correction in native proteinaceous urine, respectively, in relation to the mean of the two measurements. The mean deviation between the two methods is indicated by the red line and the 95% confidence interval is indicated by the two green lines.

A

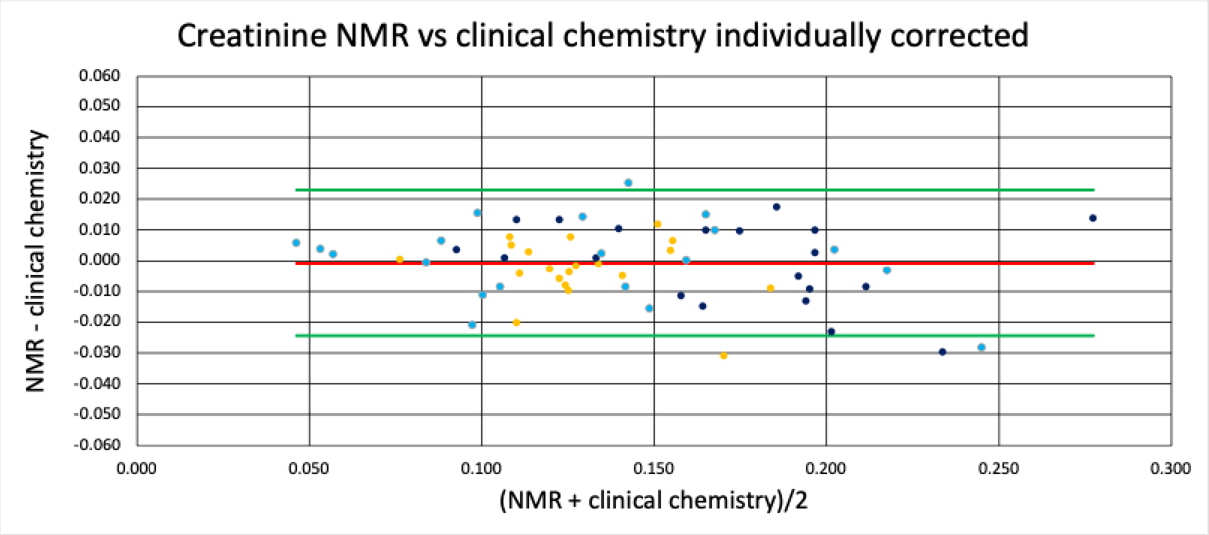

B

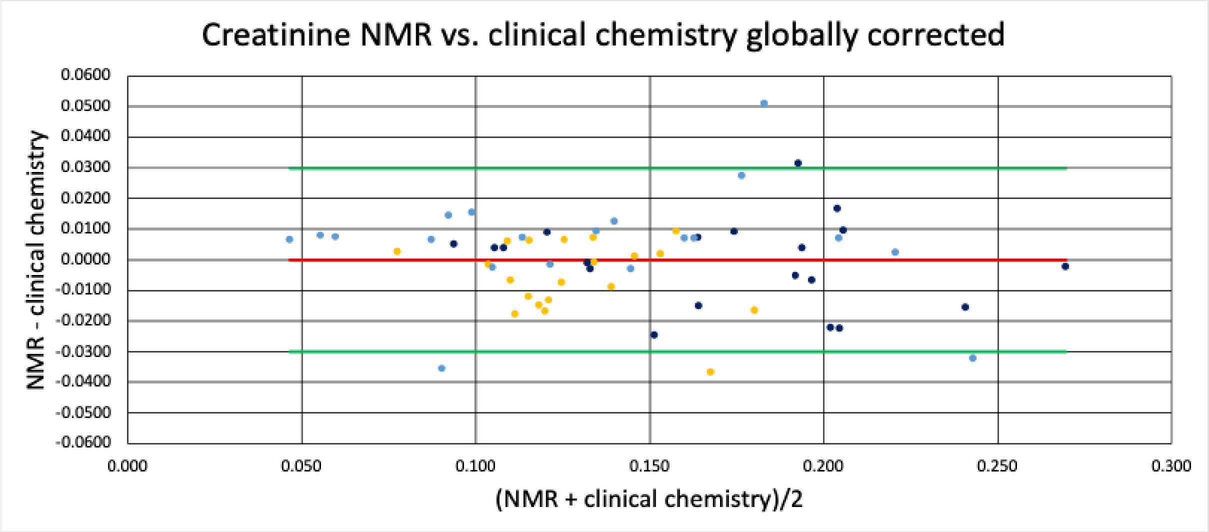

Figure S2. Differences between total plasma creatinine values obtained by NMR with (A) the application of individual linewidth-based correction factors and (B) the application of one global average linewidth-based correction factor with values from clinical chemistry. **Related to Table 1 and 2 and Figure 2.**

The mean deviation between the two methods is indicated by the red line and the 95% confidence interval by the two green lines. Specimens originating from patients with a UACR below 1.27, between 100 and 102, and above 6297 mg/g (albumin/creatinine) are color coded in orange, dark blue, and light blue, respectively.

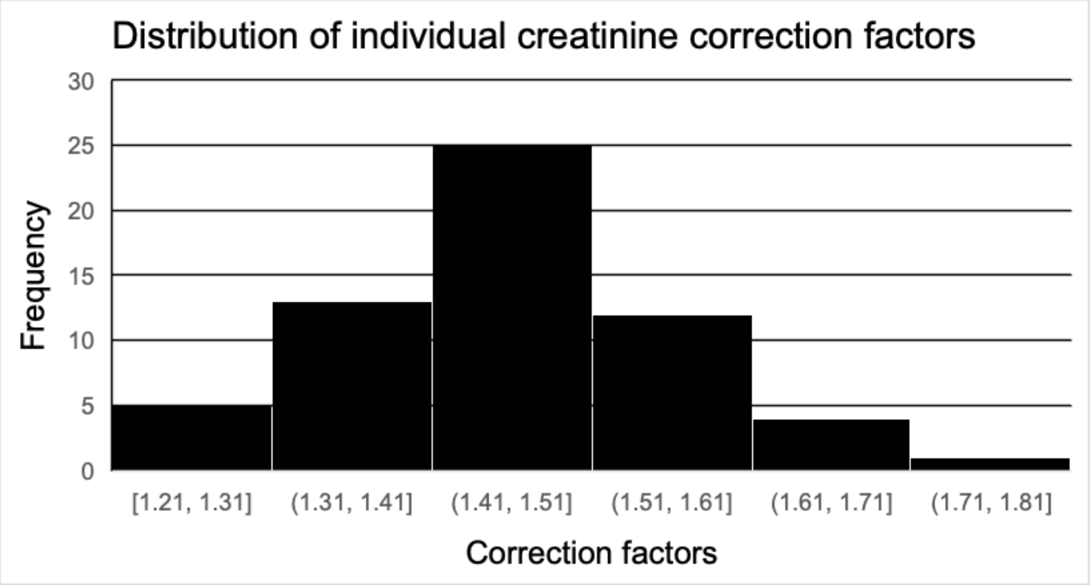

Figure S3. Distribution of individual correction factors for creatinine obtained for 60 plasma samples of the GCKD cohort. Related to Table 1 and 2 and Figure 2.

Correction factors range from 1.21 to 1.79 with a median of 1.46.

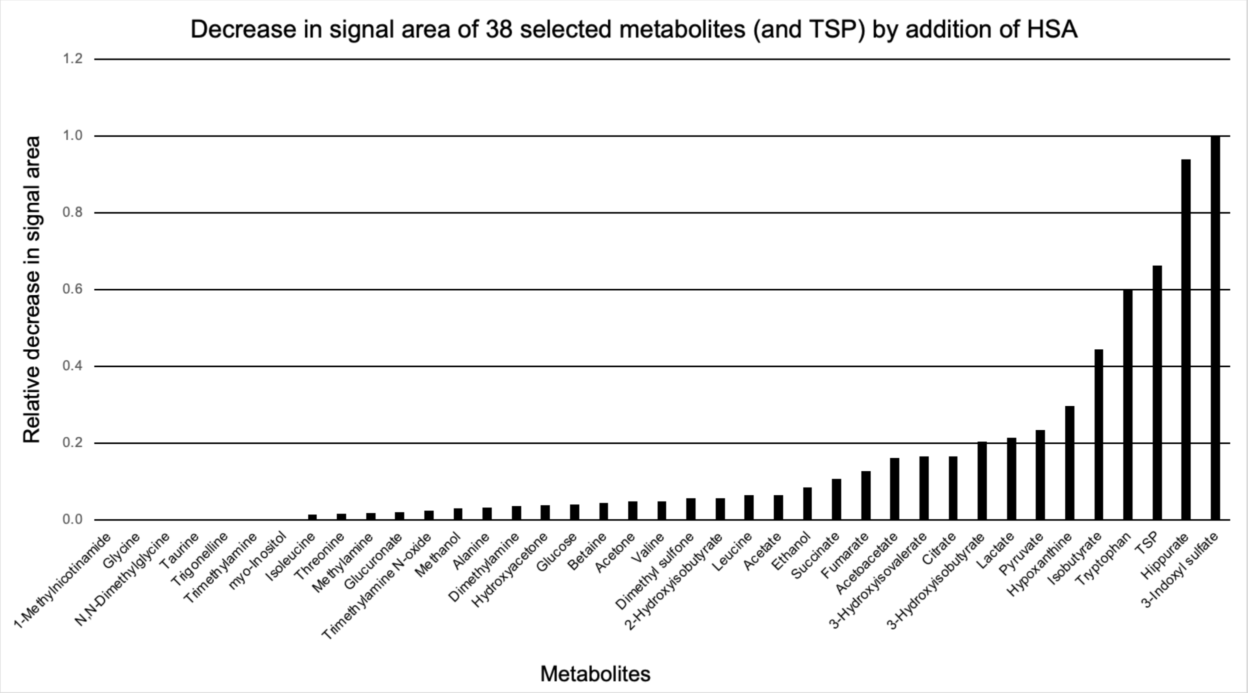

Figure S4. Differences in binding to albumin of 38 selected metabolites in a synthetic urinary matrix. Related to Figure 1.

The relative decrease in signal area in the presence of 18 g/L (0.27 mM) human serum albumin (HSA) is shown in ascending order, from the smallest to the largest decrease, for 38 metabolites and TSP. A value of one corresponds to a complete loss of signal, which reflects the complete binding of a metabolite to albumin.
