# Supplementary material for: Simultaneous determination of free and total metabolite concentrations in proteinaceous specimens by 1D 1H CPMG NMR
Source: Cell Rep Methods. 2026 Jan 16;6(1):101291. doi: 10.1016/j.crmeth.2025.101291 (PMC12853166; doi:10.1016/j.crmeth.2025.101291)
Supplement: Document S2. Article plus supplemental information [file mmc2.pdf]

Cell Reports
Methods

Report

Simultaneous determination of free and total metabolite concentrations in proteinaceous specimens by 1D ^{1}H CPMG NMR

Graphical abstract

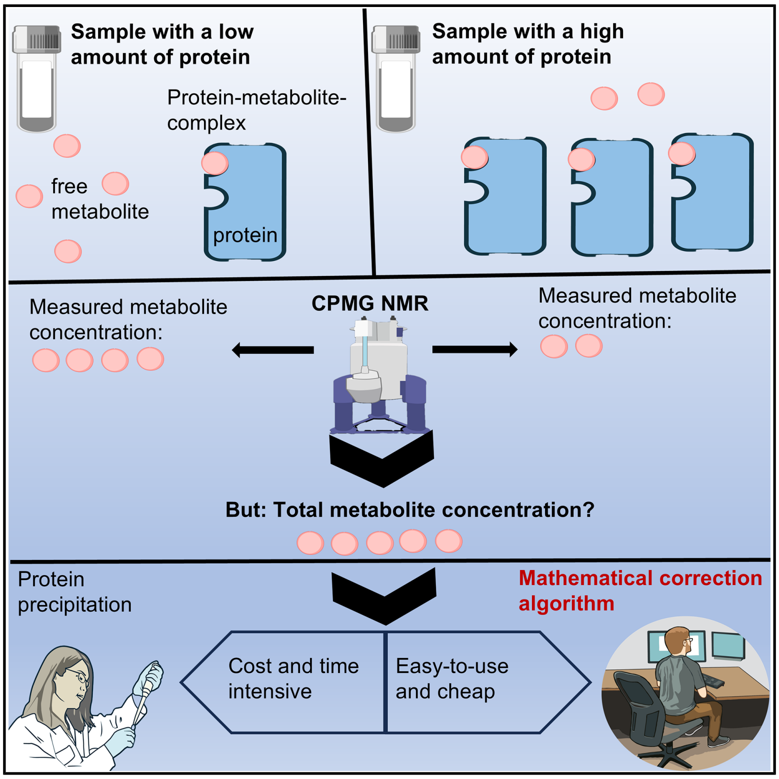

Authors

Alexander Reindl, Claudia Samol, Silke Haerteis, Helena U. Zacharias, Katja Dettmer, Peter J. Oefner, Wolfram Gronwald

Correspondence

wolfram.gronwald@ur.de

In brief

NMR spectroscopy is an important method for the study of the metabolome. Reindl et al. develop an easy-to-use tool to allow the accurate quantification of total metabolite concentrations in proteinaceous media like plasma without the need of additional experimental steps as already existing NMR spectra are utilized.

Highlights

- Easy-to-use tool for quantification of total metabolite amounts in proteinaceous media
- No need for additional experimental steps like ultrafiltration or protein precipitation
- Leaves analyzed sample intact for further investigations
- Applicable to different metabolites and different matrices like plasma or urine

Reindl et al., 2026, Cell Reports Methods 6, 101291
January 26, 2026 © 2025 The Author(s). Published by Elsevier Inc.
https://doi.org/10.1016/j.crmeth.2025.101291

CellPress

Cell Reports Methods

CellPress
OPEN ACCESS

Report

Simultaneous determination of free and total metabolite concentrations in proteinaceous specimens by 1D ^{1}H CPMG NMR

Alexander Reindl,[1] Claudia Samol,[1] Silke Haerteis,[2] Helena U. Zacharias,[3] Katja Dettmer,[1] Peter J. Oefner,[1] and Wolfram Gronwald[1,4,*]

[1]Institute of Functional Genomics, University of Regensburg, Regensburg, Germany
[2]Institute of Molecular and Cellular Anatomy, University of Regensburg, Regensburg, Germany
[3]Peter L. Reichertz Institute for Medical Informatics of TU Braunschweig and Hannover Medical School, Hannover Medical School, Hannover, Germany
[4]Lead contact
*Correspondence: wolfram.gronwald@ur.de
https://doi.org/10.1016/j.crmeth.2025.101291

MOTIVATION The investigation of the metabolome can offer new insights into medical and/or biological topics. To this end, a popular method is nuclear magnetic resonance (NMR) spectroscopy. The presence of proteins makes this analysis more challenging due to protein-metabolite interactions. Most current methods to address this problem often need additional experimental steps or are only able to quantify the unbound fraction of a specific metabolite. Therefore, we developed an approach to obtain both free and total metabolite amounts from a single NMR spectrum based on the evaluation of the individual line broadenings of the metabolite NMR signals upon protein binding.

SUMMARY

Nuclear magnetic resonance (NMR) spectroscopy is often used for the analysis of metabolites in proteinaceous biological specimens. However, the binding of metabolites to proteins impedes accurate quantitation of total metabolite concentrations by NMR, unless protein binding is disrupted by organic solvent precipitation, which increases variance and may result in the loss of volatile metabolites during post-extraction drying. Here, we present an approach for the inference of total metabolite concentrations from Carr-Purcell-Meiboom-Gill NMR spectra via computation of metabolite and sample-specific factors derived from the individual broadening of spectral peaks due to protein-metabolite binding. The method was validated on both synthetic proteinaceous samples and plasma and urine specimens including a certified reference plasma. Furthermore, results were compared with those obtained for methanol extracts of plasma specimens. In summary, our approach obviates the need for protein precipitation, is easy to use, and allows precise and reliable determination of total metabolite concentrations.

INTRODUCTION

The quantitative analysis of endogenous and exogenous small molecules, such as primary metabolites and drugs, in proteinaceous biological specimens is usually based on measuring either the total concentration (C_t) or the free concentration (C_f) of a compound of interest. The determination of total concentrations of metabolites or drugs in protein-containing specimens is typically accomplished by disruption of protein binding by organic solvents.[1,2] In contrast, most methods developed for measuring free compounds involve the separation of free fractions.[3] These approaches can be time consuming and might suffer from analyte losses.[4,5] Simultaneous determination of the free and total concentrations as well as protein-binding capacity of a compound can be accomplished by mass spectrometry under the assumption of a linear binding model and fixed stoichiometry.[6] These assumptions, however, may not apply when ligands can bind to more than one protein-binding site.[7]

NMR spectroscopy is a well-established technique for the identification and quantitation of low-molecular-weight compounds in biological fluids.[8,9] Due to its robustness and reproducibility, NMR is well suited for high-throughput analyses of large sample cohorts[10] allowing the identification of nearly 90 metabolites in human blood.[11] One drawback is the considerable signal overlap due to the large number of solutes found in biofluids. This overlap can be resolved by either

© 2025 The Author(s). Published by Elsevier Inc.
This is an open access article under the CC BY license (http://creativecommons.org/licenses/by/4.0/).

multidimensional NMR experiments[12–14] or deconvolution of overlapping signals in 1D NMR spectra by commercial (e.g., Chenomx [Chenomx Inc., Edmonton, AB, Canada]) or public-domain software such as Batman,[15] Bayesil,[16] MagMet,[17] or MetaboDecon1D.[18] Further complications arise from the presence of macromolecules such as proteins, which lead to broad background signals underneath the narrow signals of metabolites complicating their quantitation. As stated above, proteins may be removed by ultrafiltration,[19] which will also remove bound small endogenous and exogenous molecules like drugs, or by protein precipitation with typically methanol or methanol-chloroform, which will disrupt protein binding of small molecules and allow determination of their total concentrations.[20] In general, protein-metabolite interaction is a highly complex process, which plays a crucial role in cellular processes, in metabolic pathways, and also in the understanding of disease.[21] For many metabolites, protein binding has been described.[21–23] Protein precipitation, however, is also not without pitfalls and can lead to poor recovery, increased variance, and loss of volatile compounds during post-extraction drying.[24,25] Alternatively, relaxation or diffusion-based spectral editing techniques allow the suppression of macromolecular signals.[26–28] These approaches play an important role in studies where large numbers of specimens need to be compared and, thus, the experimental differences between the spectra have to be reduced to a minimum.[29] The most popular technique for the analysis of blood plasma is the Carr-Purcell-Meiboom-Gill (CPMG) pulse sequence, which employs a T_2-based relaxation filter and is recognized as an effective approach for the removal of macromolecules like proteins, while also allowing precise metabolite quantification.[30] This enables insights into topics like biomarker discovery or the identification of drug targets.[11,30] Additionally, this method has a higher reproducibility than the physical or chemical removal of macromolecules.[31] However, this relaxation filter will also attenuate the signals of protein-bound molecules. For a fixed T_2-based relaxation filter, the degree of signal attenuation depends on the degree of metabolite binding. Therefore, only the concentration of the free metabolite will be determined. To obtain the total concentration, i.e., of free and protein-bound metabolite, we have previously developed an experimental approach that provides for a given sample matrix correction factors for each metabolite by adding fixed amounts of metabolites to the sample matrix of interest followed by an analysis of the recovered amount.[32] A drawback of this approach is the need for the renewed determination of correction factors whenever the concentration and/or the composition of macromolecules changes markedly in the biological matrix under investigation.

For a metabolite in fast exchange between bound and unbound states, an average of these two states is observed, resulting in an increase in signal linewidth and a decrease in signal area as a function of the degree of protein binding. Here, we investigate how these changes in linewidth correlate with signal attenuation during the T_2-based relaxation filter. We analyze whether determination of individual linewidths of metabolite signals in each sample enables the computation of metabolite and sample-specific correction factors. These factors are solely based on already existing CPMG NMR spectra and do not require additional experimental work.

RESULTS

Development of the algorithm using signal line broadening upon protein binding

The basis of line broadening considered here is the transverse (T_2) relaxation time. Line broadening is always understood as an increase in the full width at half maximum (FWHM) signal height.

Compared to small molecules, proteins tend to have shorter T_2 relaxation times due to longer rotational correlation times and limited translational motion. Based on their longer T_2 relaxation times, small metabolites can be observed selectively by applying spin-echo loops, as implemented in the CPMG pulse sequence, prior to NMR data acquisition.[33] Thereby, the overlap with broad macromolecular signals is greatly diminished. Since bound metabolites also experience the short T_2 relaxation time of proteins, they are lost during the spin-echo cycles of the CPMG pulse sequence and only free metabolites can be subsequently quantified. Here, we introduce an algorithm for determining the total concentration of metabolites in CPMG spectra. It is based on the determination of metabolite-specific correction factors to compute the total amount from the obtained free concentration. To this end, the observed linewidth of a given metabolite is used. It is inversely proportional to the corresponding T_2 relaxation time (FWHM = $1/\pi^*T_2$). For a metabolite that is partly bound to proteins and is in fast exchange, the linewidth of the observed signal is a mixed state between the broad signal of the bound portion and the narrow signal of the free metabolite portion. Under the assumption that the linewidths of both the free and bound forms are fixed, the observed linewidth should relate to the degree of protein binding and, thereby, to the loss of detectable magnetization during the spin-echo loops of the CPMG pulse sequence. This, in turn, will allow the determination of correction factors to estimate the total concentration of a metabolite from its observed free concentration.

To this end, first the relationship between line broadening and decrease in signal area has to be established. Therefore, we initially added increasing concentrations of human serum albumin (HSA), which had not been depleted of fatty acids, to 6.025 mM of TSP (3-(trimethylsilyl)-propionic acid), which is known for its moderate binding to protein (see STAR Methods section titration of human serum albumin (HSA) for details). Figure 1A shows the linear relationship between the shim-corrected (see STAR Methods "implementation of the correction algorithm" for shim correction) TSP linewidth measured in each spectrum (y axis) and the concentration of HSA (x axis).

Next, the relationship between the TSP signal area and linewidth was investigated (Figure 1B). Note that the signal area directly corresponds to the analyte concentration in case of complete spin-lattice relaxation, requiring the use of a sufficiently long relaxation delay between scans. More specifically, the y axis in Figure 1B displays the signal-loss factor that is defined as the maximum TSP area measured in the absence of HSA divided by the TSP area obtained in the presence of HSA. The x axis displays the corresponding line broadening L_{sMb} of the TSP signal obtained for a given protein concentration (see STAR Methods for details). Fitting a linear regression between the signal-loss factor and the observed line broadening in

CellPress
OPEN ACCESS

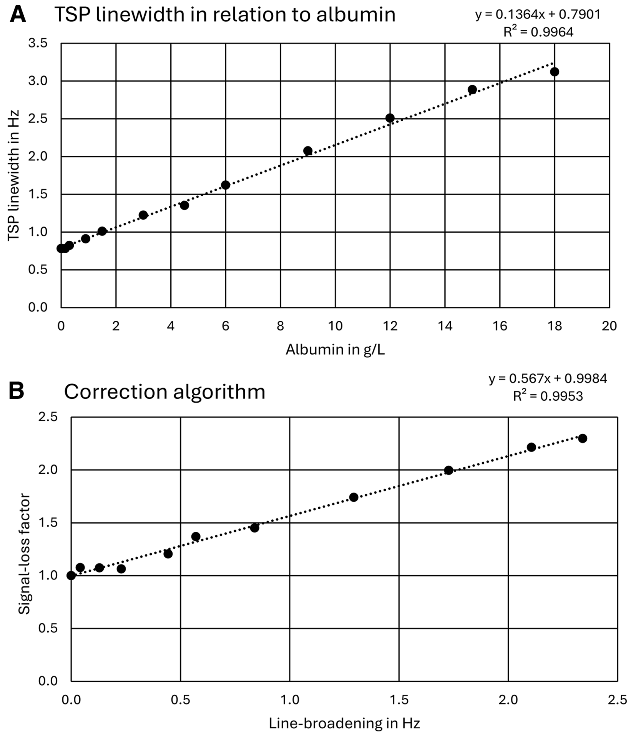

Figure 1. Development of the correction algorithm

(A) Relationship between the shim-corrected linewidth of TSP and albumin concentration.

(B) Relationship between signal-loss factors (*y* axis) and line broadening L_{sMb} of TSP. A linear correlation between line broadening and the signal-loss factor is observed. An increase in linewidth corresponds to a higher protein binding of TSP to albumin. Therefore, the free concentration of TSP decreases, which leads to a bigger signal-loss factor. This factor is then used to calculate the total concentration of TSP from its free concentration. A factor of 1 indicates no loss in signal area, hence no protein binding.

Figure 1B allows the experimental determination of the line-broadening-dependent correction factors C_b (Equation 1),

$$C_b = 0.567 \times L_{sMb} + 0.9984. \qquad \text{(Equation 1)}$$

At this point, we assume that Equation 1 holds regardless of the compound of interest. Clearly, different metabolites experience different degrees of protein binding, which will be influenced by the amount and composition of the proteins present. These aspects should be reflected in the differences of the corresponding line-broadening factors determining the correction factors. Therefore, we verify this assumption in the following. A detailed description of the developed method including a step-by-step guide is provided in the STAR Methods section implementation of the correction algorithm. Also, detailed information on the specific signals that were used for measuring the linewidths is provided in Table S1.

Validation of the algorithm in synthetic and real samples by comparison with reference methods

To validate the method, we used metabolites commonly found in plasma and urine. Not all sample matrices allowed an adequate quantification and/or use of the linewidth-based correction algorithm for each metabolite due to strong signal overlap, which varied between matrices. Furthermore, metabolites showing strong protein binding such as tryptophan, hippurate, and 3-indoxyl sulfate were detected in urine but not in plasma. Therefore, the list of analyzed metabolites is not the same in every studied specimen.

First, 11 exemplary metabolites and TSP were analyzed in the absence or presence of 18 g/L (0.27 mM) HSA (Table 1; see STAR Methods section titration of human serum albumin (HSA) for details). For 3-indoxyl sulfate and hippurate, which bind strongly to albumin, we used lower amounts of 0.9 (0.0135 mM) and 1.5 g/L (0.0226 mM) HSA, respectively. For all metabolites, individual specimens were prepared. A fixed metabolite concentration of 2.5 mM was used in all cases. For some metabolites, this concentration exceeds physiological levels. However, we chose this concentration to allow the investigation of strongly binding metabolites. Columns 2 and 3 in Table 1 show the signal areas obtained with and without the addition of HSA in relation to the internal standard formic acid, respectively. The quotient of the area values given in column 4 reflects the required correction factors to derive the total metabolite concentrations. The correction factors calculated using Equation 1 (column 5) should ideally match the values given in column 4. Therefore, the quotients of columns 4 and 5 were calculated (column 6) and found to scatter closely around 1.0, thus indicating that the linewidth-based correction given by Equation 1 allows an accurate determination of the total signal area of a given metabolite.

The algorithm was also validated on human biofluid specimens. To this end, urine and plasma specimens from the German Chronic Kidney Disease (GCKD) study[34,35] collected at baseline were employed in addition to three pooled plasma samples from acute kidney injury (AKI) patients[36] and a certified National Institute of Standards and Technology (NIST) reference plasma (see STAR Methods for details). First, the pooled plasma samples were split into 2 aliquots each. The first aliquot was left untreated, while the second aliquot was subjected to protein precipitation, as described in the section STAR Methods. The quantitative results obtained for the latter served as approximations of the total metabolite concentrations. Table 2A shows the results obtained for 16 metabolites in one of the AKI plasma pools (the results for the other two AKI plasma pools are given in Tables S2A and S2B). Note that strongly binding metabolites such as tryptophan, hippurate, and 3-indoxyl sulfate could not be detected in plasma. The second column of Table 2A contains the free metabolite concentrations determined without prior protein precipitation by 1D ^{1}H CPMG NMR, while the third column gives the total concentrations obtained for a precipitated sample. Ideally, the quotient of these two values (column 4) should equal the correction factor obtained by linewidth analysis given in column 5, as evidenced by the quotients of columns 4 and 5 listed in column 6, which scatter mostly around 1, thus indicating a good agreement between the linewidth-based correction factor and the quotient of total and free metabolite concentrations. Note that those specimens also contained 6.025 mM TSP along with the additional reference standard formic acid. Recent studies used protein-binding competitors like TSP with the goal to release the bound fraction of metabolites and, thus, obtain total metabolite concentrations.[31,37] To investigate the effect of TSP on our selected metabolites, aliquots

Table 1. Validation of linewidth-based correction factors in synthetic samples

	No albumin (2)	With albumin (3)	Area ratio (2)/(3)	Correction factor, algorithm (4)	[(2)/(3)]/(4)
Metabolite	area (a.u.)	area (a.u.)	–	–	–
3-Hydroxyisovalerate	3.566	2.975[a]	1.199	1.182	1.014
Acetoacetate	1.955	1.636[a]	1.195	1.195	1.000
Alanine	1.801	1.743[a]	1.033	1.005	1.028
Citrate	2.109	1.758[a]	1.200	1.206	0.995
Hypoxanthine	0.904	0.635[a]	1.424	1.299	1.096
Isobutyrate	3.541	1.967[a]	1.800	1.703	1.057
Lactate	2.541	1.996[a]	1.273	1.180	1.079
Pyruvate	1.234	0.944[a]	1.307	1.207	1.083
Tryptophan	0.636	0.255[a]	2.494	2.297	1.086
TSP	5.062	1.709[a]	2.962	3.256	0.910
3-Indoxyl sulphate	0.504	0.258[b]	1.953	2.039	0.958
Hippurate	1.348	0.767[c]	1.757	1.700	1.034

The table shows the signal areas of 11 metabolites and TSP, which were individually added to a synthetic matrix. Each sample contained one metabolite at a concentration of 2.5 mM.
Signal areas were determined with TopSpin 4.1.4. Metabolite signal areas were set in relation to the signal area of the internal standard formic acid, which was added to each sample at a concentration of 6 mM. The ratio of the signal areas determined without and with the addition of albumin results in a factor (area ratio (2)/(3)) required to calculate the total metabolite signal area from the signal area in the presence of protein. This factor was then compared in the last column to the correction factor determined by the linewidth-based approach. Samples were prepared with no albumin added and with the addtion of the following amounts:
[a]18 g/L (0.27 mM).
[b]0.9 g/L (0.0135 mM).
[c]1.5 g/L (0.0226 mM) HSA, resulting in a total of 24 samples.

of two of the three pooled plasma samples from AKI patients[36] were additionally ultrafiltered. The results are shown in Tables S2A and S2B. If addition of TSP releases significant amounts of bound metabolite, then the free concentrations measured in column 2 shall be higher than the filtered ones in column 7, as ultrafiltration allows only the determination of the unbound metabolite fraction. As shown in column 8 of Tables S2A and S2B, the ratios of concentrations determined by filtration and of free concentrations distribute around 1 for most metabolites, thus not supporting the release of considerable amounts of bound metabolites in our experimental setting. Also, Barrilero et al. described in their study that tryptophan, lysine, and citrate, which are also part of our study, could not be released from HSA upon the addition of TSP.[37]

Next, a certified reference plasma specimen (NIST, SRM1950, Metabolites in Frozen Human Plasma), for which total metabolite concentrations were available, was analyzed. The second and third columns of Table 2B list the free metabolite concentrations determined by 1D ^{1}H CPMG NMR and the respective NIST-certified total concentrations. Note that only metabolites with available reference values were investigated. The quotients of these two values, which represent the underestimation of the total concentration, are given in column 4, while the linewidth-based correction factors are given in column 5. As can be seen from the last column, the quotients computed from columns 4 and 5 are close to 1, indicating good agreement between the linewidth-based correction factors and the free-to-total ratios using the NIST reference values.

Furthermore, 60 plasma specimens from the GCKD cohort[34,35] with varying degree of proteinuria were selected. In total, three sets consisting of 20 specimens each were chosen. The first, second, and third sets contained specimens corresponding to a urinary albumin-to-creatinine ratio (UACR) of less than 1.27, 101.49 ± 0.77, and 6,000 to 16,000 mg/g (albumin/creatinine), respectively. For each specimen, total creatinine values were obtained by NMR with the application of individual linewidth-based correction factors and an IDMS-traceable enzymatic assay from clinical chemistry. Figure S2A shows a Bland-Altman plot comparing the two methods. On the *x* axis, the averaged values varying between 0.046 and 0.277 mM are shown, while on the *y* axis, the deviation between the two methods is given. The average deviation indicated in red amounted to 0.001 mM, and the borders of the 95% confidence interval indicated by the two green lines were at 0.023 and −0.025 mM. To investigate the effect of the specimen-specific individual correction factors in comparison to a global correction factor, the 60 individual correction factors were averaged to one global factor (Figure S2B). The average deviation between the two methods remained the same, but the borders of the 95% confidence interval increased to 0.030 and −0.030 mM. Figure S3 shows the distribution of the individual correction factors obtained for creatinine ranging from 1.21 to 1.79 with a mean value of 1.46 (median = 1.46). Further analysis of the individual correction factors for plasma creatinine with respect to the corresponding UACR showed that for UACR values < 1.25, 101.49 ± 0.77, and ≥6,000 mg/g (albumin/creatinine), averaged correction factors of 1.50, 1.47, and 1.41 were obtained, respectively.

For further validation, total concentrations of six selected amino acids that had been determined by stable-isotope dilution

Table 2. Validation of the linewidth-based correction factors on plasma samples

(A) Metabolite	Free (mM) (2)	Precipitated (mM) (3)	(3)/(2) = (4)	Algorithm (5)	(4)/(5)
Acetate	0.037	0.047	1.257	1.154	1.089
Alanine	0.246	0.298	1.210	1.133	1.068
Citrate	0.094	0.111	1.179	1.074	1.097
Creatine	0.027	0.028	1.042	1.066	0.977
Creatinine	0.074	0.090	1.225	1.252	0.978
Glucose	5.647	6.525	1.156	1.120	1.032
Glycine	0.182	0.204	1.124	1.115	1.008
Isoleucine	0.079	0.091	1.151	1.166	0.987
Lactate	1.317	1.455	1.105	1.155	0.957
Leucine	0.123	0.150	1.219	1.163	1.048
Lysine	0.081	0.110	1.355	1.395	0.971
Phenylalanine	0.050	0.069	1.370	1.417	0.967
Pyruvate	0.060	0.081	1.351	1.218	1.109
Threonine	0.112	0.128	1.145	1.086	1.055
Tyrosine	0.054	0.071	1.312	1.310	1.001
Valine	0.216	0.256	1.184	1.106	1.070
**(B) Metabolite**	**Free (mM) (2)**	**NIST (mM) (3)**	**(3)/(2)=(4)**	**Algorithm (5)**	**(4)/(5)**
Alanine	0.266	0.300 ± 0.026	1.129	1.030	1.096
Creatinine	0.047	0.060 ± 0.001	1.282	1.350	0.950
Glucose	4.520	4.560 ± 0.056	1.009	1.000	1.009
Glycine	0.244	0.245 ± 0.016	1.005	1.050	0.957
Isoleucine	0.052	0.056 ± 0.003	1.078	1.035	1.041
Leucine	0.084	0.100 ± 0.006	1.190	1.080	1.101
Threonine	0.117	0.120 ± 0.006	1.022	1.000	1.022
Valine	0.170	0.182 ± 0.010	1.069	1.030	1.038
Tyrosine	0.044	0.057 ± 0.003	1.291	1.240	1.041
Lysine	0.072	0.140 ± 0.014	1.950	1.890	1.032
Phenylalanine	0.034	0.051 ± 0.007	1.504	1.655	0.909

(A) On a pooled AKI plasma.
(B) On a certified reference plasma.

liquid chromatography-tandem mass spectrometry (LC-MS/MS) in methanol extracts of 5 random baseline plasma specimens of the GCKD cohort[34,35] were compared with those derived from 1D ^{1}H CPMG NMR spectra using sample-specific linewidth-based correction factors (Tables S3). In each table, the second column lists the free metabolite concentrations obtained by 1D ^{1}H CPMG NMR, followed by the individual linewidth-based correction factor and the total concentration of each metabolite. The latter should ideally reflect the value obtained by LC-MS/MS given in the next column. Also, the quotients of MS and free NMR values in column 6 should approximate the linewidth-based correction factors. The agreement between LC-MS/MS and corrected NMR values given by the quotient of the respective values is shown in the last column of Table S3. Table S4 summarizes the sample- and metabolite-specific correction factors for the five GCKD plasma specimens together with the mean (±SD) of the metabolite-specific correction factors across the 5 samples. The last column shows for each metabolite the average agreement in concentrations between MS and corrected NMR data as a ratio of the two.

Next, urine specimens of GCKD patients[34,35] with varying degrees of proteinuria ranging from 0.84 to 15,783.59 mg/g (albumin/creatinine) were investigated. The aim was to test how well linewidth-based estimation of total metabolite concentrations copes with different amounts of protein in urine. In total, 15 pooled specimens were analyzed (see STAR Methods for details). We focused on three metabolites that showed particularly strong protein binding, namely, 3-indoxyl sulfate, hippurate, and tryptophan. For these metabolites, the acquired signals are so broad that they are not amenable to quantification by Chenomx. Therefore, we compared total signal areas as determined by TopSpin 4.1.4. Figure 2 shows a Bland-Altman plot of differences between the peak area integrals for total tryptophan determined by 1D ^{1}H CPMG NMR in methanol extracts of urine and indirectly in native urine specimens by linewidth-based estimation, respectively, versus the mean of the two measurements. The corresponding results for 3-indoxyl sulfate and hippurate are given in Figures S1A and S1B, respectively. The 95% limits of agreement between the two methods as indicated by the upper and lower green lines, were in all cases below 10% of

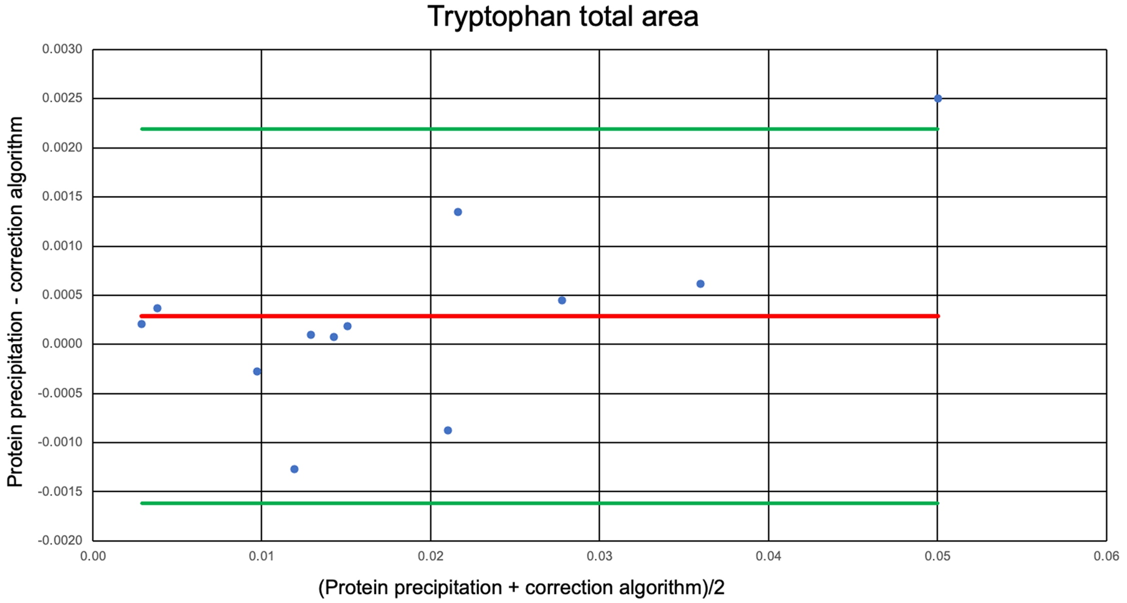

Figure 2. Comparison of the total areas of tryptophan in urine specimens determined by the linewidth-based correction algorithm versus protein precipitation

The Bland-Altman plot shows the differences between the peak area integrals for total tryptophan determined by 1D ^{1}H CPMG NMR after protein precipitation of urine with methanol and the use of linewidth-based correction in native proteinaceous urine, respectively, in relation to the mean of the two measurements. The mean deviation between the two methods is indicated by the red line, and the 95% confidence interval is indicated by the two green lines.

the average signal areas. For all three metabolites, these data showed only small and no systematic differences between the two methods. Note that although 15 specimens were investigated, not all spectra allowed an adequate determination of all the above-mentioned metabolites, resulting in 12 values for tryptophan, 11 for hippurate, and 9 for 3-indoxyl sulfate, respectively.

Differences in binding of 38 selected metabolites to HSA

Besides developing a new linewidth-based algorithm for deriving total metabolite concentrations from 1D ^{1}H CPMG NMR spectra of proteinaceous biological specimens, we were interested in qualitatively analyzing the binding of 38 selected metabolites to HSA. For each metabolite present at a concentration of 2.5 mM, the signal area with no HSA addition was compared to that obtained after addition of 18 g/L (0.27 mM) HSA in artificially generated samples as described in STAR Methods. As mentioned above, this metabolite concentration does not necessarily represent the expected concentrations of the selected metabolites in human plasma, but this fixed concentration allowed a direct comparison of the selected metabolites with each other. To this end, data were acquired in all cases with a CPMG pulse sequence as detailed in STAR Methods. In Figure S4, metabolites were ranked according to their loss in signal area upon addition of HSA. A particular strong binding to HSA was observed for 3-indoxyl sulfate, hippurate, the reference compound TSP, and tryptophan, while almost no HSA binding was observed for 3-methylnicotinamide, glycine, N,N-dimethylglycine, taurine, trigonelline, trimethylamine, and myo-inositol. All signal areas were determined by TopSpin 4.1.4. Note that although we had investigated the metabolites creatine and creatinine, we excluded them from Figure S4 as they were part of the artificial matrix used to simulate a natural environment (see STAR Methods for details) and, thus, had different conditions in terms of binding competition compared to the other 38 metabolites.

DISCUSSION

We presented a method to compute total metabolite concentrations from free concentration values determined by 1D ^{1}H CPMG NMR by employing correction factors that are individually determined for metabolites based on the broadening of their respective NMR signals by protein binding.

The proposed algorithm was developed based on an experimentally determined relationship between loss in signal area and increase in linewidth of the TSP reference signal (Figure 1B; Equation 1). This correction algorithm was first tested on an exemplary set of 11 typical metabolites (Table 1). Ideally, the ratio of the quantitation results obtained with and without the addition of HSA should correspond to the correction factors obtained by the algorithm. As evident from Table 1, observed deviations ranged from 0.0% for acetoacetate to 9.6% for hypoxanthine, indicating that the approach is applicable in these cases.

Next, the algorithm was tested under more physiological conditions and with different matrices. We started by investigating 16 metabolites in three human plasma pools (Tables 2A, S2A, and S2B). Compared to the results obtained after protein precipitation, as shown in Table 2A for the first pooled plasma sample, the results obtained by application of the algorithm to a 1D ^{1}H CPMG NMR spectrum of unprecipitated plasma differed

only slightly from 0.1% for tyrosine to 10.9% for pyruvate (column 6 of Table 2A). For the two additional pooled plasma samples, comparable results were obtained. These results support the ability of the algorithm to reliably derive total metabolite concentrations of proteinaceous biological specimens.

The algorithm was further validated against the total concentration values of six amino acids and creatinine, which had been determined in 5 plasma specimens by stable-isotope dilution LC-MS/MS and an IDMS-traceable enzymatic assay, respectively (Table S3). As can be seen from the last columns of Tables S3 and S4, the quotients between LC-MS/MS (or enzymatic assay) and corrected NMR values scattered around 1, further corroborating the overall good performance of the linewidth-based method.

In addition, we analyzed total creatinine values in 60 plasma specimens from patients with varying degrees of proteinuria ranging from 0.84 to 15,784 mg/g (urinary albumin/creatinine) and corresponding serum albumin values ranging from 46.19 to 10.10 (g/L). First, individual correction factors were determined for each specimen resulting in good agreement with results from clinical chemistry (Figure S2A) with an average deviation of 6.9%. Employing one global correction factor for all creatinine values slightly decreased the agreement (Figure S2B) with an averaged deviation of 8.3%. The subtle benefit of using individual correction factors is also explained by their distribution ranging from 1.21 to 1.79 (Figure S3).

For further validation, reference plasma, for which certified total concentrations for 11 metabolites were available, was analyzed (Table 2B). Ideally, the ratio between free metabolite concentrations and certified reference values should equal the linewidth-based correction factors. Here, the obtained correction factors differed from the certified values within a range of 0.9% for glucose to 10.1% for leucine.

Next, the applicability to urine was investigated. Here, we concentrated on strong binding compounds, namely, tryptophan, 3-indoxyl sulfate, and hippurate, due to the low amounts of total protein (less than 150 mg/g creatinine) present in urine specimens of healthy people. Note that in case of proteinuria, high amounts of protein in excess of 500 mg/g may be found in urine. Here we studied urine containing up to 15,783.59 mg/g (albumin/creatinine). Figure 2 shows good agreement and no significant bias between estimated signal areas of total urinary tryptophan applying linewidth-based correction to 1D ^{1}H CPMG NMR spectra of native urine specimens and signal areas obtained for methanol extracts of the very same specimens. The same held true for 3-indoxyl sulfate and hippurate (Figures S1A and S1B). Therefore, the algorithm is also suitable for strong-binding metabolites as long as they are detected.

As in our previous study, in which we performed spike-in experiments with known amounts of metabolites in a pool of 100 randomly selected plasma samples from the GCKD study to facilitate determination of total metabolite concentrations using 1D ^{1}H CPMG NMR,[32] we see significant differences in plasma protein binding between different metabolites. Moreover, we also observe significant differences in plasma protein binding between different plasma specimens. Therefore, the calculation of total metabolite concentrations with the help of one metabolite-specific correction factor derived from a plasma pool is only appropriate for samples where plasma protein concentrations and competition of metabolites for protein-binding sites are mostly constant across samples, which, however, is often not the case. This is confirmed for creatinine in Figures S2 and S3.

In summary, our algorithm enables the user to estimate total metabolite concentrations in proteinaceous biological samples without any extra experimental steps.

Limitations of the study

Limitations include possible difficulties in measuring the linewidth of multiplets and of signals with a non-zero baseline. Also, in the presence of strong signal overlap the accuracy of the determined linewidth may be compromised. Furthermore, compounds such as branched-chain amino acids may show inherent flexibility even in the bound state, which will impact (decrease) the linewidth. Also, strongly binding compounds may not be detected in 1D ^{1}H CPMG NMR spectra of highly proteinaceous specimens. Further note that the derived correction algorithm is solely based on experimental observations where we see a linear relationship between the exponentially decaying NMR signal and the linearly increasing linewidth. This indicates that for the examples shown, we are in good approximation in the initial linear phase of the decaying NMR signals.

RESOURCE AVAILABILITY

Lead contact

Requests for further information and resources should be directed to and will be fulfilled by the lead contact, Wolfram Gronwald (wolfram.gronwald@ur.de).

Materials availability

This study did not generate new unique reagents.

Data and code availability

- All data reported in this paper will be shared by the lead contact upon reasonable request.
- This paper does not report original code.
- Any additional information required to reanalyze the data reported in this paper is available from the lead contact upon reasonable request.

ACKNOWLEDGMENTS

The authors acknowledge the support from the Deutsche Forschungsgemeinschaft (DFG, German Research Foundation), project number 509149993, TRR 374. H.U.Z. was supported by the German Federal Ministry of Research, Technology and Space (BMFTR) within the framework of the e:Med research and funding concept (grant number 01ZX1912A). We thank all the GCKD study participants for their time and important contributions, all participating nephrologists' practices and outpatient clinics for their continued support, as well as the GCKD study personnel and investigators for their enormous commitment. We would also like to thank all GCKD investigators, which are as follows: Kai-Uwe Eckardt, Heike Meiselbach, Markus P. Schneider, Mario Schiffer, Hans-Ulrich Prokosch, Barbara Bärthlein, Andreas Beck, André Reis, Arif B. Ekici, Susanne Becker, Ulrike Alberth-Schmidt, Anke Weigel, Sabine Marschall, Gerd Walz, Anna Köttgen, Ulla T. Schultheiβ, Fruzsina Kotsis, Simone Meder, Erna Mitsch, Ursula Reinhard, Jürgen Floege, Rafael Kramann, Turgay Saritas, Elke Schaeffner, Seema Baid-Agrawal, Kerstin Theisen, Kai Schmidt-Ott, Martin Zeier, Claudia Sommerer, Mehtap Aykac, Gunter Wolf, Martin Busch, Andy Steiner, Thomas Sitter, Christoph Wanner, Vera Krane, Britta Bauer, Florian Kronenberg, Barbara Kollerits, Lukas Forer, Julia Raschenberger, Sebastian Schönherr, Hansi Weissensteiner, Peter J. Oefner, Wolfram Gronwald, Matthias Schmid, and Jennifer Nadal.

AUTHOR CONTRIBUTIONS

Conceptualization, P.J.O. and W.G.; methodology, A.R. and W.G.; software, W.G.; investigation, A.R., C.S., K.D., and W.G.; formal analysis, A.R., P.J.O., and W.G.; data curation, A.R., C.S., K.D., and W.G.; writing – original draft, A.R., H.U.Z., and W.G.; writing – review & editing, P.J.O.; visualization, A.R. and W.G.; funding acquisition, K.D., P.J.O., and W.G.; resources, P.J.O., H.U.Z., S.H., and W.G.; supervision, P.J.O. and W.G. All authors have read and agreed to the published version of the manuscript.

DECLARATION OF INTERESTS

The authors declare no competing interests.

STAR★METHODS

Detailed methods are provided in the online version of this paper and include the following:

- KEY RESOURCES TABLE
- EXPERIMENTAL MODEL AND STUDY PARTICIPANT DETAILS
- METHOD DETAILS
  - Protein precipitation
  - Ultrafiltration of plasma
  - Titration of human serum albumin (HSA)
  - NMR spectroscopy
- QUANTIFICATION AND STATISTICAL ANALYSIS
  - NMR quantification and linewidth determination
  - Liquid chromatography−Tandem mass spectrometry (LC-MS/MS) quantification
  - Implementation of the correction algorithm
  - Lower limits of quantification

SUPPLEMENTAL INFORMATION

Supplemental information can be found online at https://doi.org/10.1016/j.crmeth.2025.101291.

Received: July 21, 2025
Revised: November 11, 2025
Accepted: December 11, 2025
Published: January 16, 2026

REFERENCES

1. Rico, E., González, O., Blanco, M.E., and Alonso, R.M. (2014). Evaluation of human plasma sample preparation protocols for untargeted metabolic profiles analyzed by UHPLC-ESI-TOF-MS. Anal. Bioanal. Chem. *406*, 7641–7652. https://doi.org/10.1007/s00216-014-8212-y.
2. McHugh, C.E., Flott, T.L., Schooff, C.R., Smiley, Z., Puskarich, M.A., Myers, D.D., Younger, J.G., Jones, A.E., and Stringer, K.A. (2018). Rapid, Reproducible, Quantifiable NMR Metabolomics: Methanol and Methanol: Chloroform Precipitation for Removal of Macromolecules in Serum and Whole Blood. Metabolites *8*, 93. https://doi.org/10.3390/metabo8040093.
3. Seyfinejad, B., Ozkan, S.A., and Jouyban, A. (2021). Recent advances in the determination of unbound concentration and plasma protein binding of drugs: Analytical methods. Talanta *225*, 122052. https://doi.org/10.1016/j.talanta.2020.122052.
4. Nagana Gowda, G.A., Gowda, Y.N., and Raftery, D. (2015). Massive glutamine cyclization to pyroglutamic acid in human serum discovered using NMR spectroscopy. Anal. Chem. *87*, 3800–3805. https://doi.org/10.1021/ac504435b.
5. Musteata, F.M. (2011). Monitoring free drug concentrations: challenges. Bioanalysis *3*, 1753–1768. https://doi.org/10.4155/bio.11.187.
6. Cibotaru, D., Celestin, M.N., Kane, M.P., and Musteata, F.M. (2021). Method for Simultaneous Determination of Free Concentration, Total Concentration, and Plasma Binding Capacity in Clinical Samples. J. Pharm. Sci. *110*, 1401–1411. https://doi.org/10.1016/j.xphs.2020.12.001.
7. Jayaraj, A., Schwanz, H.A., Spencer, D.J., Bhasin, S., Hamilton, J.A., Jayaram, B., Goldman, A.L., Krishna, M., Krishnan, M., Shah, A., et al. (2021). Allosterically Coupled Multisite Binding of Testosterone to Human Serum Albumin. Endocrinology *162*, bqaa199. https://doi.org/10.1210/endocr/bqaa199.
8. Holmes, E., Foxall, P.J., Spraul, M., Farrant, R.D., Nicholson, J.K., and Lindon, J.C. (1997). 750 MHz 1H NMR Spectroscopy Characterisation of the Complex Metabolic Pattern of Urine from Patients with Inborn Errors of Metabolism: 2-hydroxyglutaric Aciduria and Maple Syrup Urine Disease. J. Pharm. Biomed. Anal. *15*, 1647–1659. https://doi.org/10.1016/s0731-7085(97)00066-6.
9. Nicholson, J.K., Foxall, P.J., Spraul, M., Farrant, R.D., and Lindon, J.C. (1995). 750 MHz 1H and 1H-13C NMR Spectroscopy of Human Blood Plasma. Anal. Chem. *67*, 793–811. https://doi.org/10.1021/ac00101a004.
10. Dumas, M.E., Maibaum, E.C., Teague, C., Ueshima, H., Zhou, B., Lindon, J.C., Nicholson, J.K., Stamler, J., Elliott, P., Chan, Q., and Holmes, E. (2006). Assessment of Analytical Reproducibility of 1H NMR Spectroscopy Based Metabonomics for Large-Scale Epidemiological Research: the INTERMAP Study. Anal. Chem. *78*, 2199–2208. https://doi.org/10.1021/ac0517085.
11. Nagana Gowda, G.A., Pascua, V., and Raftery, D. (2022). A new limit for blood metabolite analysis using 1H NMR spectroscopy. J. Magn. Reson. Open *12–13*, 100082. https://doi.org/10.1016/j.jmro.2022.100082.
12. Gronwald, W., Klein, M.S., Kaspar, H., Fagerer, S.R., Nürnberger, N., Dettmer, K., Bertsch, T., and Oefner, P.J. (2008). Urinary Metabolite Quantification Employing 2D NMR Spectroscopy. Anal. Chem. *80*, 9288–9297. https://doi.org/10.1021/ac801627c.
13. Schlippenbach, T.V., Oefner, P.J., and Gronwald, W. (2018). Systematic Evaluation of Non-Uniform Sampling Parameters in the Targeted Analysis of Urine Metabolites by 1H,1H 2D NMR Spectroscopy. Sci. Rep. *8*, 4249. https://doi.org/10.1038/s41598-018-22541-0.
14. Weitzel, A., Samol, C., Oefner, P.J., and Gronwald, W. (2020). Robust Metabolite Quantification from J-Compensated 2D 1H-13C-HSQC Experiments. Metabolites *10*, 449. https://doi.org/10.3390/metabo10110449.
15. Hao, J., Astle, W., De Iorio, M., and Ebbels, T.M.D. (2012). BATMAN–an R package for the automated quantification of metabolites from nuclear magnetic resonance spectra using a Bayesian model. Bioinformatics *28*, 2088–2090. https://doi.org/10.1093/bioinformatics/bts308.
16. Ravanbakhsh, S., Liu, P., Bjorndahl, T.C., Mandal, R., Grant, J.R., Wilson, M., Eisner, R., Sinelnikov, I., Hu, X., Luchinat, C., et al. (2015). Accurate, fully-automated NMR spectral profiling for metabolomics. PLoS One *10*, e0124219. https://doi.org/10.1371/journal.pone.0124219.
17. Rout, M., Lipfert, M., Lee, B.L., Berjanskii, M., Assempour, N., Fresno, R.V., Cayuela, A.S., Dong, Y., Johnson, M., Shahin, H., et al. (2023). MagMet: A fully automated web server for targeted nuclear magnetic resonance metabolomics of plasma and serum. Magn. Reson. Chem. *61*, 681–704. https://doi.org/10.1002/mrc.5371.
18. Häckl, M., Tauber, P., Schweda, F., Zacharias, H.U., Altenbuchinger, M., Oefner, P.J., and Gronwald, W. (2021). An R-Package for the Deconvolution and Integration of 1D NMR Data: MetaboDecon1D. Metabolites *11*, 452. https://doi.org/10.3390/metabo11070452.
19. Klein, M.S., Almstetter, M.F., Schlamberger, G., Nürnberger, N., Dettmer, K., Oefner, P.J., Meyer, H.H.D., Wiedemann, S., and Gronwald, W. (2010). Nuclear magnetic resonance and mass spectrometry-based milk metabolomics in dairy cows during early and late lactation. J. Dairy Sci. *93*, 1539–1550. https://doi.org/10.3168/jds.2009-2563.
20. Nagana Gowda, G.A., Gowda, Y.N., and Raftery, D. (2015). Expanding the limits of human blood metabolite quantitation using NMR spectroscopy. Anal. Chem. *87*, 706–715. https://doi.org/10.1021/ac503651e.

21. Kurbatov, I., Dolgalev, G., Arzumanian, V., Kiseleva, O., and Poverennaya, E. (2023). The Knowns and Unknowns in Protein-Metabolite Interactions. Int. J. Mol. Sci. *24*, 4155. https://doi.org/10.3390/ijms24044155.

22. Diether, M., Nikolaev, Y., Allain, F.H., and Sauer, U. (2019). Systematic mapping of protein-metabolite interactions in central metabolism of Escherichia coli. Mol. Syst. Biol. *15*, e9008. https://doi.org/10.15252/msb.20199008.

23. Piazza, I., Kochanowski, K., Cappelletti, V., Fuhrer, T., Noor, E., Sauer, U., and Picotti, P. (2018). A Map of Protein-Metabolite Interactions Reveals Principles of Chemical Communication. Cell *172*, 358–372.e23. https://doi.org/10.1016/j.cell.2017.12.006.

24. Tiziani, S., Emwas, A.H., Lodi, A., Ludwig, C., Bunce, C.M., Viant, M.R., and Günther, U.L. (2008). Optimized metabolite extraction from blood serum for 1H nuclear magnetic resonance spectroscopy. Anal. Biochem. *377*, 16–23. https://doi.org/10.1016/j.ab.2008.01.037.

25. Sedgwick, G.W., Fenton, T.W., and Thompson, J.R. (1991). Effect of protein precipitating agents on the recovery of plasma free amino acids. Can. J. Anim. Sci. *71*, 953–957. https://doi.org/10.4141/cjas91-116.

26. Tang, H., Wang, Y., Nicholson, J.K., and Lindon, J.C. (2004). Use of relaxation-edited one-dimensional and two dimensional nuclear magnetic resonance spectroscopy to improve detection of small metabolites in blood plasma. Anal. Biochem. *325*, 260–272. https://doi.org/10.1016/j.ab.2003.10.033.

27. Liu, M., Nicholson, J.K., and Lindon, J.C. (1996). High-resolution diffusion and relaxation edited one- and two-dimensional 1H NMR spectroscopy of biological fluids. Anal. Chem. *68*, 3370–3376. https://doi.org/10.1021/ac960426p.

28. Kriat, M., Confort-Gouny, S., Vion-Dury, J., Sciaky, M., Viout, P., and Cozzone, P.J. (1992). Quantitation of metabolites in human blood serum by proton magnetic resonance spectroscopy. A comparative study of the use of formate and TSP as concentration standards. NMR Biomed. *5*, 179–184. https://doi.org/10.1002/nbm.1940050404.

29. Grasso, D., Pillozzi, S., Tazza, I., Bertelli, M., Campanacci, D.A., Palchetti, I., and Bernini, A. (2022). An improved NMR approach for metabolomics of intact serum samples. Anal. Biochem. *654*, 114826. https://doi.org/10.1016/j.ab.2022.114826.

30. Madrid-Gambin, F., Oller, S., Marco, S., Pozo, Ó.J., Andres-Lacueva, C., and Llorach, R. (2023). Quantitative plasma profiling by 1H NMR-based metabolomics: impact of sample treatment. Front. Mol. Biosci. *10*, 1125582. https://doi.org/10.3389/fmolb.2023.1125582.

31. Vanhove, K., Derveaux, E., Mesotten, L., Thomeer, M., Criel, M., Mariën, H., and Adriaensens, P. (2022). Unraveling the Rewired Metabolism in Lung Cancer Using Quantitative NMR Metabolomics. Int. J. Mol. Sci. *23*, 5602. https://doi.org/10.3390/ijms23105602.

32. Wallmeier, J., Samol, C., Ellmann, L., Zacharias, H.U., Vogl, F.C., Garcia, M., Dettmer, K., Oefner, P.J., and Gronwald, W.; GCKD Study Investigators (2017). Quantification of Metabolites by NMR Spectroscopy in the Presence of Protein. J. Proteome Res. *16*, 1784–1796. https://doi.org/10.1021/acs.jproteome.7b00057.

33. Wang, Y., Bollard, M.E., Keun, H., Antti, H., Beckonert, O., Ebbels, T.M., Lindon, J.C., Holmes, E., Tang, H., and Nicholson, J.K. (2003). Spectral editing and pattern recognition methods applied to high-resolution magic-angle spinning 1H nuclear magnetic resonance spectroscopy of liver tissues. Anal. Biochem. *323*, 26–32. https://doi.org/10.1016/j.ab.2003.07.026.

34. Eckardt, K.U., Bärthlein, B., Baid-Agrawal, S., Beck, A., Busch, M., Eitner, F., Ekici, A.B., Floege, J., Gefeller, O., Haller, H., et al. (2012). The German Chronic Kidney Disease (GCKD) study: design and methods. Nephrol. Dial. Transpl. *27*, 1454–1460. https://doi.org/10.1093/ndt/gfr456.

35. Titze, S., Schmid, M., Köttgen, A., Busch, M., Floege, J., Wanner, C., Kronenberg, F., and Eckardt, K.U.; GCKD study investigators (2015). Disease burden and risk profile in referred patients with moderate chronic kidney disease: composition of the German Chronic Kidney Disease (GCKD) cohort. Nephrol. Dial. Transpl. *30*, 441–451. https://doi.org/10.1093/ndt/gfu294.

36. Zacharias, H.U., Hochrein, J., Vogl, F.C., Schley, G., Mayer, F., Jeleazcov, C., Eckardt, K.U., Willam, C., Oefner, P.J., and Gronwald, W. (2015). Identification of Plasma Metabolites Prognostic of Acute Kidney Injury after Cardiac Surgery with Cardiopulmonary Bypass. J. Proteome Res. *14*, 2897–2905. https://doi.org/10.1021/acs.jproteome.5b00219.

37. Barrilero, R., Ramírez, N., Vallvé, J.C., Taverner, D., Fuertes, R., Amigó, N., and Correig, X. (2017). Unravelling and Quantifying the "NMR-Invisible" Metabolites Interacting with Human Serum Albumin by Binding Competition and T2 Relaxation-Based Decomposition Analysis. J. Proteome Res. *16*, 1847–1856. https://doi.org/10.1021/acs.jproteome.6b00814.

38. Beckonert, O., Keun, H.C., Ebbels, T.M.D., Bundy, J., Holmes, E., Lindon, J.C., and Nicholson, J.K. (2007). Metabolic Profiling, Metabolomic and Metabonomic Procedures for NMR Spectroscopy of Urine, Plasma, Serum and Tissue Extracts. Nat. Protoc. *2*, 2692–2703. https://doi.org/10.1038/nprot.2007.376.

39. van der Goot, A.T., Zhu, W., Vázquez-Manrique, R.P., Seinstra, R.I., Dettmer, K., Michels, H., Farina, F., Krijnen, J., Melki, R., Buijsman, R.C., et al. (2012). Delaying aging and the aging-associated decline in protein homeostasis by inhibition of tryptophan degradation. Proc. Natl. Acad. Sci. USA *109*, 14912–14917. https://doi.org/10.1073/pnas.1203083109.

STAR★METHODS

KEY RESOURCES TABLE

REAGENT or RESOURCE	SOURCE	IDENTIFIER
Biological samples		
Urine samples	GCKD study[34,35]	N/A
Plasma samples	GCKD study,[34,35] NIST (SRM1950), Zacharias et al.[36]	N/A
Sigmatrix Urine Diluent (synthetic negative urine control)	Merck (Sigma-Aldrich)	Cat#SAE0074
Chemicals, peptides, and recombinant proteins		
1-Methylnicotinamide iodide	Toronto Research Chemicals	Cat#M323235
α - Hydroxyisobutyric acid	Merck (Sigma-Aldrich)	Cat#164976
Sodium β - hydroxyisobutyric acid	Merck (Sigma-Aldrich)	Cat#36105
β - Hydroxyisovaleric acid	Merck (Sigma-Aldrich)	Cat#55453
3 – Indoxyl sulfate (potassium – 3 – indoxyl sulfate potassium salt)	Merck (Sigma-Aldrich)	Cat#I3875
Lithium acetoacetic acid	Honeywell Fluka	Cat#00478
Acetone	Merck (Sigma-Aldrich)	Cat#176800010
L - Alanine	Merck (Sigma-Aldrich)	Cat#05129
Succinic acid	Merck (Sigma-Aldrich)	Cat#S3674
Betaine	Merck (Sigma-Aldrich)	Cat#61962
Sodium Pyruvic acid	Merck (Sigma-Aldrich)	Cat#P8574
Citric acid monohydrate	Merck (Sigma-Aldrich)	Cat#1.00244.0500
Creatine	Merck (Sigma-Aldrich)	Cat#C0780
Creatinine anhydrous	Merck (Sigma-Aldrich)	Cat#C4255
Dimethylamine hydrochloride	Merck (Sigma-Aldrich)	Cat#126365
Dimethyl sulfone	Merck (Sigma-Aldrich)	Cat#M81705
Acetic acid	VWR International GmbH	Cat#49199
Ethanol	Merck (Sigma-Aldrich)	Cat#32205
Fumaric acid	Merck (Sigma-Aldrich)	Cat#F8509
(D+) - Glucose	Carl Roth GmbH + Co. KG	Cat#X997.2
D – Glucuronic acid	Merck (Sigma-Aldrich)	Cat#71560
Glycine	Merck Chemicals GmbH	Cat#K40355601944
Hippuric acid	Merck (Sigma-Aldrich)	Cat#112003
Hydroxyacetone	Merck (Sigma-Aldrich)	Cat#138185
Hypoxanthine	Merck (Sigma-Aldrich)	Cat#56700
Isobutyric acid	Merck (Sigma-Aldrich)	Cat#58360
DL - Isoleucine	Merck (Sigma-Aldrich)	Cat#58884
DL - Leucine	Merck (Sigma-Aldrich)	Cat#61840
Methanol	VWR International GmbH	Cat#9822.2500GL
Methylamine	Merck (Sigma-Aldrich)	Cat#8.22091.1000
DL – Lactic acid	Merck (Sigma-Aldrich)	Cat#69785
Myo-inositol	Merck (Sigma-Aldrich)	Cat#I5125
N,N - Dimethylglycine	Merck (Sigma-Aldrich)	Cat#D1156
Taurine	Merck (Sigma-Aldrich)	Cat#T8691
L - Threonine	Merck (Sigma-Aldrich)	Cat#89180
Trigonelline hydrochloride	Merck (Sigma-Aldrich)	Cat#T5509
Trimethylamine	Merck (Sigma-Aldrich)	Cat#W324108-SAMPLE-K
Trimethylamine N-oxide	N/A	N/A

(*Continued on next page*)

Continued

REAGENT or RESOURCE	SOURCE	IDENTIFIER
DL - Tryptophan	Merck (Sigma-Aldrich)	Cat#93680
DL - Valine	Merck (Sigma-Aldrich)	Cat#94640
3 - Methylxanthine	Merck (Sigma-Aldrich)	Cat#222526
Acetamide	Merck (Sigma-Aldrich)	Cat#00160
Theophylline	Merck (Sigma-Aldrich)	Cat#T1633
Adenine	Merck (Sigma-Aldrich)	Cat#01830
1,7 - Dimethylxanthine	Merck (Sigma-Aldrich)	Cat#D5385
Xanthine	Merck (Sigma-Aldrich)	Cat#95490
L – Ascorbic acid	Merck Supelco (Sigma-Aldrich)	Cat#R600408
Caffeine	Merck (Sigma-Aldrich)	Cat#C0750
3-(trimethylsilyl)-propionic acid-2,2,3,3-d_4 sodium salt (TSP)	Carl Roth GmbH + Co. KG	Cat#9922.1
Formic acid	Merck Supelco (Sigma-Aldrich)	Cat#5.33002.0050
di-Potassium hydrogen phosphate	Carl Roth GmbH + Co. KG	Cat#P749.2
Potassium dihydrogen phosphate	Merck Supelco (Sigma-Aldrich)	Cat#1.04873.1000
Deuterium oxide	Merck (Sigma-Aldrich)	Cat#151882
Nicotinic acid	Merck (Sigma-Aldrich)	Cat#72309
Albumin from human serum, lyophilized powder, $\geq$96% (agarose gel electrophoresis, remainder mostly globulins, not depleted of fatty acids), molecular weight: 66478 g/mol	Merck (Sigma-Aldrich)	Cat#A1653
Amino acid mixture	Merck (Sigma-Aldrich)	AAS18-5mL
13C/15N-labeled canonical amino acid mix	Eurisotop (CIL)	MSK-CAA-1
Ammoniumformate	Merck (Sigma-Aldrich)	516961
HFBA	Merck (Sigma-Aldrich)	52411-25ML-F
Ethylacetate	Avantor (VWR)	85481.320
Methanol	Avantor (VWR)	85800.320
Isooctane	Avantor (VWR)	28781.325
Propylchloroformate	Merck (Sigma-Aldrich)	249467
n-Propanol	Merck (Sigma-Aldrich)	1.01024.1000
3-Picoline	Sigma-Aldrich	236276
**Software and algorithms**		
TopSpin 3.1 and 4.1.4	Bruker	https://www.bruker.com/en/products-and-solutions/mr/nmr-software/topspin.html
Chenomx NMR Suite 8.3	Chenomx	https://www.chenomx.com
AMIX - Viewer 3.9.13	Bruker	https://www.bruker.com/en/products-and-solutions/mr/nmr-software/amix.html
**Other**		
600 MHz Bruker Avance III Spectrometer	Bruker	N/A
Certified 5 mm NMR Tubes, 4″	CortecNet	Cat#Z172599
Amicon Ultra-4, PLGC Ultracel-PL membrane, 10 kDa	Merck Millipore	Cat#UFC801096

EXPERIMENTAL MODEL AND STUDY PARTICIPANT DETAILS

Urine specimens were collected at the start of the German Chronic Kidney Disease (GCKD) study.[34,35] This prospective observational study aims at understanding the causes, course and risk factors of progressive kidney insufficiency. Starting in 2009, it enrolled a total of 5217 CKD patients, 18 to 74 years of age, with an estimated glomerular filtration rate (eGFR) of 30–60 mL/min/1.73 m^2, or an eGFR of >60 mL/min/1.73 m^2 and one of the following four albuminuria/proteinuria indications: urine albumin/urine creatinine >300 mg/g, urine albumin >300 mg/day, total urine protein/urine creatinine >500 mg/g, or total urine protein >500 mg/day. Exclusion criteria included active cancer, previous transplants, or heart failure stage NYHA (New York Heart Association) IV. Informed consent was

obtained from all patients. The study was carried out in accordance with the Declaration of Helsinki, registered in the German Register of Clinical Trials (DRKS 00003971), and approved by the ethics committees of the participating institutions.[34,35] The native urine specimens were stored at −80°C until measurement.

Plasma specimens originated from the GCKD study,[34,35] the National Institute of Standards and Technology (NIST, Gaithersburg, MD, USA SRM1950, Metabolites in Frozen Human Plasma), and a study on acute kidney injury (AKI) after cardiac surgery.[36] The AKI study was approved by the local Institutional Review Board (Ethik-Kommission der Medizinischen Fakultät der Friedrich-Alexander-Universität Erlangen-Nürnberg, #4010), reference: https://doi.org/10.1371/journal.pone.0145042. All plasma samples were stored at −80°C until measurement. Samples were thawed for sample preparation and then directly measured without any further freezing and thawing.

METHOD DETAILS

Protein precipitation

108 urine specimens from the GCKD study[34,35] were assigned to five groups according to their urine albumin-to-creatinine ratio (UACR; mg/g). Group 1 comprised participants with UACR values between 0.84 and 1.29 mg/g, while UACR values in groups 2, 3, 4, and 5 ranged from 29.5–30.46 mg/g, 293.1–306.65 mg/g, 2878.21–3104.54 mg/g, and 7749.48–15783.59 mg/g, respectively. Groups 1 to 4 consisted of 25 participants each, group 5 of 8 participants. From each group, 9 (8 from group 5) participants were selected: 3 with the highest urine creatinine levels within the group, 3 with urine creatinine levels close to the median, and 3 or 2 (group 5) participants with the lowest urine creatinine levels in the respective group. Samples selected on the basis of their urine creatinine values were then pooled, resulting in 15 pooled samples out of a total of 44 urine specimens. Thereby, we aimed to evaluate the effect of protein binding in urine specimens differing in total solute content. From each pooled specimen, 400 μL aliquots were taken that served either as an unprecipitated reference sample or were subjected to protein precipitation. Before precipitation, 10 μL of 80 mM nicotinic acid were added as an extraction standard to correct for loss of non-volatile metabolites during extraction. Next, 1.6 mL of 100% methanol were added to achieve a ratio of 80% methanol to 20% urinary sample. The total volume of 2 mL was thoroughly vortexed and centrifuged for 5 min at 4°C and 10,000 g. The supernatants were transferred to fresh vials. The remaining sample was washed with 200 μL of 80% methanol, followed by thorough mixing and centrifugation for 5 min at 4°C and 10,000 g. Supernatants were removed and combined with the first ones. The washing step was repeated one more time with the difference that the centrifugation was performed at 12,000 g. The combined supernatants were then evaporated to complete dryness using a vacuum evaporator (CombiDancer, Hettich AG, Bäch, Switzerland) and reconstituted in 400 μL of pure water (ELGA LabWater, Celle, Germany).

The three pooled plasma samples from the AKI study[36] were precipitated according to the protocol by Gowda et al.[20] Briefly, 800 μL of methanol were added to 400 μL of plasma, thoroughly mixed, incubated for 20 min at −20°C, and centrifuged for 30 min at 13,400 g. Supernatants were transferred to fresh vials, evaporated to complete dryness, and reconstituted in 400 μL of pure water. Two of those pooled plasma samples were also ultrafiltrated as described below.

Ultrafiltration of plasma

For ultrafiltration of the pooled plasma samples, we used a filter with a cutoff of 10 kDa. The centrifugal filters were washed with 3 mL of water and centrifuged at 4,000 g for 30 min. Then, 4 mL of each of the 2 plasma samples together with 20 μL of 80 mM nicotinic acid as extraction standard were added to a filter tube and centrifuged at 4,000 g for 60 min.

Titration of human serum albumin (HSA)

Individual stock solutions of 38 metabolites and TSP were prepared in pure water at a concentration of 10 mM. Then, 100 μL of the metabolite standard were individually added to 200 μL of synthetic urine (Sigmatrix Urine Diluent, containing $CaCl_2$, $MgCl_2$, KCl, NaCl, NaH_2PO_4, Na_2SO_4, urea, creatinine, creatine and sodium azide as a preservative) to simulate a natural environment. For the results presented in Figure S4, 100 μL HSA were added to each of these mixtures to reach a final HSA concentration of 18 g/L, while keeping the metabolite or TSP concentration constant at 2.5 mM. Matching samples without the addition of HSA were prepared by replacing the HSA solution with pure water, resulting in a total of 78 samples. For the results presented in Table 1, 10 pairs of these samples were selected. For 3-indoxyl sulfate and hippurate (Table 1) we used lower amounts of HSA (0.9 and 1.5 g/L) to allow for proper signal detection despite high protein binding. For the development of the correction algorithm itself (Figure 1; Equation 1), HSA was added in 14 different concentrations (see Table. Added HSA concentrations) to TSP, which was kept at a concentration of 6.025 mM.

Table. Added HSA concentrations.

sample number	albumin concentration [g/L]	albumin concentration [μmol/L]
1	0.000	0
2	0.015	0.226

(*Continued on next page*)

Continued

sample number	albumin concentration [g/L]	albumin concentration [µmol/L]
3	0.030	0.451
4	0.150	2.256
5	0.300	4.513
6	0.900	13.538
7	1.500	22.564
8	3.000	45.128
9	4.500	67.692
10	6.000	90.255
11	9.000	135.383
12	12.000	180.511
13	15.000	225.639
14	18.000	270.766

NMR spectroscopy

Plasma and urine specimens

400 µL of sample were mixed with 200 µL of 0.1 M phosphate buffer (pH 7.4), 50 µL of 0.75% (w) TSP-2,2,3,3-d4 in deuterium oxide (D2O) and 10 µL of a 240 mM stock solution of formic acid. For quantification we used formic acid as internal standard. Unlike TSP, it does not bind to protein, which makes it an adequate reference also in highly proteinaceous specimens like plasma. Although being an endogenous metabolite, its normal concentrations in plasma are several orders of magnitude lower than the used reference concentration. Therefore, the error introduced by the endogenous amount of formic acid is neglectable.[29] Additionally, 3.9 mM of borate was added to urinary samples to prevent bacterial growth.

Titration of HSA

For the titration experiments with HSA, 400 µL of the above-described respective protein-metabolite mixtures were mixed with 250 µL of 0.1 M phosphate buffer containing 50 µL D2O and the internal reference formic acid at a final concentration of 6.0 mM.

All NMR experiments were performed on a Bruker Avance III HD 600 MHz spectrometer, equipped with a Bruker SampleJet, using a triple resonance (^{1}H, ^{13}C, ^{15}N, ^{2}H lock) helium cooled cryoprobe with z-gradient. Tuning and matching of the probe as well as locking and shimming of the sample were performed automatically. The CPMG pulse sequence was used to suppress macromolecular signals.[38] 1D ^{1}H CPMG spectra were acquired as described previously.[32] In short, 128 scans with 72k data points each were acquired with an acquisition time and T_1 relaxation delay of 3.07 and 4 s per scan, respectively, at a temperature of 298 K or 310 K for pooled plasma. The spectral width was set to 12019 Hz. The total echo time of the CPMG cycle was adjusted to 80 ms, employing a τ_e of 300 µs, 128 repetitions and an approximate 180° pulse-length of 26 µs. All spectra were processed with TopSpin 4.1.4. Data were Fourier transformed, phase corrected, applying a line broadening of 0.3 Hz and zero-filled to 128k data points resulting in a resolution of 0.09 Hz per point. All spectra were baseline corrected by application of a polynomial baseline correction.

QUANTIFICATION AND STATISTICAL ANALYSIS

NMR quantification and linewidth determination

To identify the exact signal positions of the selected metabolites, a total of 65 spike-in experiments were conducted. For quantitation of the unbound metabolite fractions featured in Tables 2A, 2B, and S3: Figure S2 and S3, the Chenomx NMR Suite 8.3 (Chenomx Inc., Edmonton, Alberta, Canada) was used in a semi-automated fashion employing the results from the spike-in experiments described above for signal selection. For the determination of the quantification results featured in Table 1, in Figures 2, S1, and S4 and for the development of the algorithm (Figure 1), signal areas were acquired by integration with TopSpin 4.1.4, also using the information from the above-mentioned spike-in experiments. All quantitative results were acquired in relation to the added internal reference standard formic acid. For quantitation, all signals of a metabolite were thoroughly evaluated, albeit considering primarily well resolved signals in regions with no or only limited signal overlap. Linewidths were measured automatically using TopSpin 4.1.4. For isolated signals the command “peakw” that measures the full linewidth at half height was applied. For crowded regions deconvolution was applied with the command “dcon”. Linewidths were manually controlled for reliability.

Liquid chromatography−Tandem mass spectrometry (LC-MS/MS) quantification

For the determination of total concentrations of amino acids by LC−MS/MS, baseline plasma specimens of the GCKD cohort were precipitated with methanol, before derivatization of amino acids with propyl-chloroformate/propanol using 10 µL of plasma extract and subsequent analysis as previously described.[39]

Implementation of the correction algorithm

The FWHM is used as a measure of the linewidth of a peak in an NMR spectrum. The FWHM of a given compound depends not only on protein binding, but also on other factors, in particular temperature and homogeneity of the magnetic field. Although adequate, thorough and constant calibration of these settings is highly important to allow a comparison of different spectra, variations can occur especially with respect to the homogeneity of the magnetic field. Therefore, a standard compound for shim correction must be added to the sample. We used formic acid, as it does not bind to protein.[29] Therefore, its linewidth will only be affected by measurement dependent factors such as temperature and shim. By comparison of the actual linewidth of formic acid in the sample to the formic acid linewidth of a standard reference spectrum, a measurement specific shim correction can be computed by calculating the difference of those two values. This shim-correction is then applied to the linewidths of all metabolites of interest in the analyzed spectrum as described below under "Shim correction". The standard linewidth of formic acid was experimentally derived from a protein-free well-shimmed reference sample.

The application of the algorithm is described in the following for a metabolite M, where M may be any detectable metabolite of interest.

1. Linewidth determination: First, the optimal linewidths of all metabolites of interest, L_{oM}, and that of formic acid, L_{oF}, which is used for shim correction, are determined in a protein-free environment, ensuring optimal shimming of the samples. Table S1 lists these values along with the specific signals and chemical shifts for 29 exemplary metabolites (including formic acid) and TSP that were obtained by means of a Bruker Avance III HD spectrometer at 298 K. Whenever possible we used well-shaped signals showing little signal overlap for the determination of linewidths. For aromatic metabolites, such as hippurate, multiplets were selected in the well resolved aromatic region to avoid the generally more crowded aliphatic regions of the spectra. Second, the linewidths of formic acid (L_{sF}) and the specific metabolites (L_{sM}) are determined in the sample(s) of interest.
2. Shim correction: Subtract the reference linewidth of formic acid, L_{oF}, from the formic acid linewidth in a spectrum of interest, L_{sF}, to obtain the shim correction S_c (L_{sF}-L_{oF} = S_c). This shim correction S_c is then subtracted from all metabolite-specific linewidths in the spectrum of interest, L_{sM}, to obtain the shim-corrected linewidths, L_{sMc}.
3. Line broadening of M due to protein binding: Subtract the optimal line width L_{oM} of M from the corresponding shim corrected linewidth L_{sMc} in sample s to obtain the protein line-broadening difference L_{sMb} of that metabolite in the specific sample (L_{sMc}-L_{oM} = L_{sMb}).
4. Correction factor: Insert the L_{sMb} value in Equation 1 to compute the correction factor C_b.

Example: M = TSP, L_{oM} of TSP = 0.78 Hz, L_{oF} of formic acid = 0.70 Hz.

1. Measure the linewidths of formic acid (L_{sF}) and TSP (L_{sM}) in the spectrum of interest:

example: L_{sF} = 0.80 Hz, L_{sM} = 1.50 Hz.

2. Shim correction: subtract L_{oF} from L_{sF}: 0.8 Hz–0.7 Hz = 0.1 Hz (S_c), correct the measured linewidth L_{sM}; for TSP: 1.5 Hz–0.1 Hz = 1.4 Hz (L_{sMc}).
3. Calculate line broadening: subtract L_{oM} from L_{sMc}; for TSP: 1.4 Hz–0.78 Hz = 0.62 Hz.
4. Insert the line broadening difference L_{sMb} of TSP in Equation 1 to obtain the correction factor C_b: 0.567*0.62 + 0.9984 = 1.35 (C_b);

thus, the quantified (unbound) concentration of TSP must be multiplied by 1.35 to obtain the total TSP concentration, example: unbound TSP concentration: 4.45 mM - > multiply by 1.35.

4.45mM * 1.35 = 6.008 mM, the total concentration of TSP in the sample.

Lower limits of quantification

To apply the correction algorithm successfully a reliable determination of the free metabolite concentrations is required. The lower limit of quantification (LLOQ) was determined using triplicate measurements of a series of geometrically diluted standard solutions. The LLOQ was defined as the lowest analyte concentration exhibiting a relative standard deviation between measurements of less than 20%. For creatine, the least abundant metabolite presented in Table 2A an LLOQ of 0.005 mM was determined. This corresponds to a signal-to-noise ratio of 30.25 for the creatine signal at 3.04 ppm. For other metabolites similar LLOQ values ranging from 0.001 mM to 0.01 mM were obtained. Please note that these values depend, among others, on the used spectrometer, the used probe (here a very sensitive He-cooled cryo-probe was used), and the number of scans. In practical applications concerning biofluids such as urine or plasma, quantification is usually not limited by the LLOQ or insufficient signal-to-noise ratio but by signal overlap with other metabolites and background signals.

Cell Reports Methods, Volume *6*

Supplemental information

Simultaneous determination of free and total metabolite concentrations in proteinaceous specimens by 1D ^{1}H CPMG NMR

Alexander Reindl, Claudia Samol, Silke Haerteis, Helena U. Zacharias, Katja Dettmer, Peter J. Oefner, and Wolfram Gronwald

Table S1. NMR characteristics of model compounds and their optimal linewidths L_{oM} in the absence of protein (29 metabolites and TSP). Related to STAR Methods subheading "IMPLEMENTATION OF THE CORRECTION ALGORITHM".

Note that for multiplets with highly overlapping signals the width of the whole multiplet measured at half height will be used. All linewidths were experimentally determined in pure water at 298 K.

metabolite	chemical shift and atoms[1]	signal characteristic	optimal linewidth L_{oM} [Hz]
3-Hydroxyisovalerate	2.4 ppm, $(\mathbf{H}2)_2$	singlet	1.39
3-Indoxyl sulfate	7.7 ppm, (**H**4)	full multiplet	10.42
Acetate	1.9 ppm, $(\mathbf{H}2)_3$	singlet	0.74
Acetoacetate	2.3 ppm, $(\mathbf{H}4)_3$	singlet	1.47
Acetone	2.2 ppm, $(\mathbf{H}1)_3$, $(\mathbf{H}3)_3$	singlet	0.73
Alanine	1.5 ppm, $(\mathbf{H}3)_3$	left peak of doublet	1.03
Citrate	2.7 ppm, (**H**2B)	left peak of doublet	1.25
Creatine	3.0 ppm, $(\mathbf{H}5)_3$	singlet	0.96
Creatinine	4.0 ppm, $(\mathbf{H}2)_2$	singlet	0.96
Ethanol	1.2 ppm, $(\mathbf{H}2)_3$	middle peak of triplet	0.75
Formate	8.4 ppm, (**H**1)	singlet	0.70
Glucose	3.5 ppm, (**H**3-b)	singlet	1.22
Glycine	3.6 ppm, $(\mathbf{H}2)_2$	singlet	0.77
Hippurate	7.8 ppm, (**H**6, **H**10)	full multiplet	10.46
Hypoxanthine	8.2 ppm, (**H**8)	left peak	1.40
Isobutyrate	1.1 ppm, $(\mathbf{H}3)_3$, $(\mathbf{H}4)_3$	both peaks together	7.82
Isoleucine	1.0 ppm, $(\mathbf{H}6)_3$	left peak of doublet	1.18
Lactate	1.3 ppm, $(\mathbf{H}3)_3$	left peak of doublet	0.87
Leucine	1.0 ppm, $(\mathbf{H}5)_3$, $(\mathbf{H}6)_3$	middle peak of multiplet	1.76
Lysine	3.0 ppm, $(\mathbf{H}6)_2$	middle peak of triplet	1.92
Methanol	3.4 ppm, $(\mathbf{H}1)_3$	singlet	0.72
N,N-Dimethylglycine	2.9 ppm, $(\mathbf{H}4)_3$, $(\mathbf{H}5)_3$	singlet	0.87
Phenylalanine	7.3 ppm, (**H**5), (**H**9)	full multiplet	9.98
Pyruvate	2.4 ppm, $(\mathbf{H}3)_3$	singlet	0.73
Succinate	2.4 ppm, $(\mathbf{H}2)_2$, $(\mathbf{H}3)_2$	singlet	0.80
Threonine	3.6 ppm, (**H**2)	right peak of doublet	0.89
Tryptophan	7.7 ppm, (**H**9)	full multiplet	10.38
TSP	0.0 ppm, $(\mathbf{H}5)_3$, $(\mathbf{H}6)_3$, $(\mathbf{H}7)_3$	singlet	0.78
Tyrosine	6.9 ppm, (**H**6), (**H**8)	full multiplet	9.60
Valine	1.0 ppm, $(\mathbf{H}4)_3$	left peak of doublet	1.11

[1]All atoms were named according to the nomenclature provided by the Bruker BBIOREFCODE database (Bruker BioSpin GmbH, Ettlingen, Germany).

Table S2. Validation of the linewidth-based correction factors on plasma pools. Related to Table 2.

The table compares the correction factors obtained by the new algorithm with those from protein precipitation. (A) Plasma pool no. 2. (B) Plasma pool no. 3. All concentrations were determined by NMR quantification. Column 7 shows the concentrations of the metabolites after ultrafiltration of the sample and column 8 relates those concentrations to the free metabolite concentrations. Here, for most metabolites the quotients of filtrated and free concentration scatter around 1.0. Note that Table S2A does not include threonine as it could not be adequately quantified in this spectrum. Instead, values for succinate are shown, which could not be sufficiently quantified in the spectra of Tables 2A and S2B.

(A) metabolite	free [mM] (2)	precipitated [mM] (3)	(3)/(2)=(4)	algorithm (5)	(4)/(5)	filtrated [mM] (7)	(7)/(2)
Acetate	0.166	0.193	1.165	1.071	1.088	0.171	1.033
Alanine	0.157	0.179	1.146	1.114	1.029	0.165	1.051
Citrate	0.134	0.131	0.975	1.001	0.974	0.123	0.915
Creatine	0.021	0.021	1.029	1.028	1.001	0.019	0.942
Creatinine	0.064	0.080	1.238	1.224	1.011	0.069	1.079
Glucose	6.615	6.941	1.049	1.052	0.997	6.276	0.949
Glycine	0.121	0.135	1.115	1.098	1.015	0.095	0.783
Isoleucine	0.043	0.047	1.112	1.034	1.075	0.039	0.920
Lactate	1.027	1.063	1.035	1.106	0.936	0.986	0.960
Leucine	0.086	0.098	1.143	1.123	1.017	0.079	0.920
Lysine	0.085	0.093	1.092	1.157	0.944	0.089	1.040
Phenylalanine	0.043	0.050	1.164	1.191	0.977	0.035	0.812
Pyruvate	0.072	0.082	1.132	1.140	0.993	0.089	1.234
Succinate	0.015	0.019	1.224	1.257	0.974	0.016	1.072
Tyrosine	0.037	0.045	1.203	1.146	1.050	0.041	1.102
Valine	0.174	0.200	1.155	1.079	1.071	0.175	1.008
(B) metabolite	free [mM] (2)	precipitated [mM] (3)	(3)/(2)=(4)	algorithm (5)	(4)/(5)	filtrated [mM] (7)	(7)/(2)
Acetate	0.070	0.086	1.225	1.214	1.009	0.075	1.064
Alanine	0.239	0.283	1.188	1.146	1.037	0.256	1.074
Citrate	0.151	0.161	1.069	1.029	1.039	0.140	0.929
Creatine	0.037	0.044	1.200	1.095	1.096	0.037	1.005
Creatinine	0.083	0.108	1.302	1.298	1.003	0.092	1.102
Glucose	6.918	7.381	1.067	1.092	0.977	6.344	0.917
Glycine	0.141	0.161	1.149	1.144	1.004	0.107	0.764
Isoleucine	0.058	0.068	1.164	1.210	0.962	0.067	1.146
Lactate	1.539	1.632	1.061	1.191	0.891	1.618	1.052
Leucine	0.112	0.139	1.242	1.191	1.043	0.113	1.008
Lysine	0.087	0.110	1.257	1.180	1.065	0.108	1.236
Phenylalanine	0.046	0.061	1.335	1.331	1.003	0.054	1.175
Pyruvate	0.092	0.110	1.202	1.197	1.005	0.119	1.301
Threonine	0.090	0.090	1.003	1.023	0.980	0.094	1.047
Tyrosine	0.052	0.073	1.404	1.316	1.067	0.062	1.191
Valine	0.208	0.244	1.172	1.106	1.060	0.218	1.049

Table S3. Comparison of the linewidth-based correction algorithm with LC-MS/MS and an IDMS-traceable enzymatic assay in five different plasma specimens. Related to Table 2.
Validation of the total concentrations of selected amino acids and creatinine determined by application of linewidth-based correction factors against the absolute concentrations measured either by an IDMS-traceable enzymatic assay (creatinine) or by stable-isotope dilution LC-MS/MS. (A) plasma specimen 1. (B) plasma specimen 2. (C) plasma specimen 3. (D) plasma specimen 4. (E) plasma specimen 5.

(A) metabolite	NMR, free conc. [mM] (2)	correction factor (3)	NMR, total conc. [mM] (4)	MS, total conc. [mM] (5)	(5/2)	(5/4)
Alanine	0.188	1.28	0.240	0.249	1.33	1.04
Creatinine	0.065	1.26	0.082	0.088	1.36	1.08
Glycine	0.204	1.12	0.228	0.237	1.16	1.04
Isoleucine	0.038	1.09	0.041	0.047	1.24	1.14
Threonine	0.111	1.05	0.117	0.116	1.04	0.99
Tyrosine	0.037	1.34	0.049	0.049	1.33	0.99
Valine	0.156	0.99	0.155	0.186	1.19	1.20
(B) metabolite	NMR, free conc. [mM] (2)	correction factor (3)	NMR, total conc. [mM] (4)	MS, total conc. [mM] (5)	(5/2)	(5/4)
Alanine	0.212	1.21	0.256	0.266	1.26	1.04
Creatinine	0.077	1.51	0.117	0.126	1.63	1.08
Glycine	0.113	1.32	0.149	0.167	1.48	1.12
Isoleucine	0.029	1.04	0.030	0.041	1.42	1.38
Threonine	0.085	1.04	0.088	0.087	1.03	0.99
Tyrosine	0.055	1.36	0.075	0.075	1.36	1.00
Valine	0.153	1.03	0.158	0.168	1.10	1.07
(C) metabolite	NMR, free conc. [mM] (2)	correction factor (3)	NMR, total conc. [mM] (4)	MS, total conc. [mM] (5)	(5/2)	(5/4)
Alanine	0.191	1.07	0.204	0.236	1.24	1.16
Creatinine	0.084	1.30	0.109	0.126	1.50	1.15
Glycine	0.141	1.21	0.171	0.194	1.38	1.14
Isoleucine	0.034	1.01	0.035	0.041	1.20	1.18
Threonine	0.067	0.92	0.062	0.074	1.10	1.19
Tyrosine	0.031	1.28	0.040	0.041	1.32	1.03
Valine	0.134	1.03	0.137	0.148	1.11	1.08
(D) metabolite	NMR, free conc. [mM] (2)	correction factor (3)	NMR, total conc. [mM] (4)	MS, total conc. [mM] (5)	(5/2)	(5/4)
Alanine	0.282	1.07	0.303	0.333	1.18	1.10
Creatinine	0.141	1.40	0.197	0.210	1.49	1.06
Glycine	0.092	1.13	0.104	0.129	1.40	1.25
Isoleucine	0.044	1.11	0.049	0.057	1.30	1.17
Threonine	0.070	1.02	0.071	0.075	1.08	1.05
Tyrosine	0.035	1.28	0.045	0.048	1.37	1.08
Valine	0.155	1.03	0.160	0.168	1.09	1.05
(E) metabolite	NMR, free conc. [mM] (2)	correction factor (3)	NMR, total conc. [mM] (4)	MS, total conc. [mM] (5)	(5/2)	(5/4)
Alanine	0.196	1.14	0.222	0.233	1.19	1.05
Creatinine	0.096	1.36	0.131	0.145	1.50	1.11
Glycine	0.146	1.15	0.168	0.164	1.13	0.98
Isoleucine	0.055	1.05	0.058	0.068	1.24	1.18
Threonine	0.075	1.22	0.091	0.079	1.06	0.87
Tyrosine	0.028	1.35	0.038	0.038	1.34	0.99
Valine	0.194	1.02	0.198	0.200	1.03	1.01

Table S4. Linewidth-based correction factors for six selected amino acids and creatinine in five different plasma specimens – summary. Related to Table 2.

Shown are the metabolite-specific linewidth-based correction factors obtained for each of the five GCKD plasma specimens (Table S3) and the mean (±SD) of the metabolite-specific correction factors obtained for the five specimens. The last column gives for each metabolite the average agreement in concentrations between MS and corrected NMR data as a ratio of the two.

metabolite	sample 1	sample 2	sample 3	sample 4	sample 5	mean±SD	mean MS vs mean corrected NMR data
Alanine	1.28	1.21	1.07	1.07	1.14	1.15±0.09	1.08
Creatinine	1.26	1.51	1.30	1.40	1.36	1.37±0.10	1.10
Glycine	1.12	1.32	1.21	1.13	1.15	1.19±0.08	1.11
Isoleucine	1.09	1.04	1.01	1.11	1.05	1.06±0.04	1.21
Threonine	1.05	1.04	0.92	1.02	1.22	1.05±0.11	1.02
Tyrosine	1.34	1.36	1.28	1.28	1.35	1.32±0.04	1.02
Valine	0.99	1.03	1.03	1.03	1.02	1.02±0.02	1.08

A

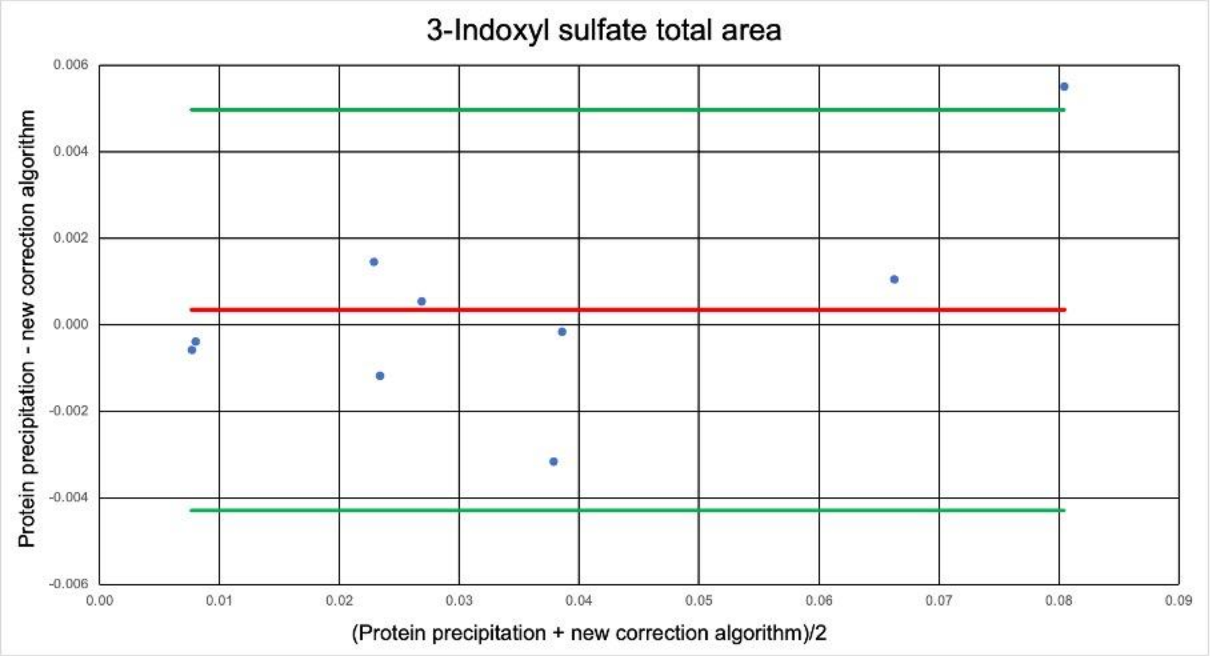

B

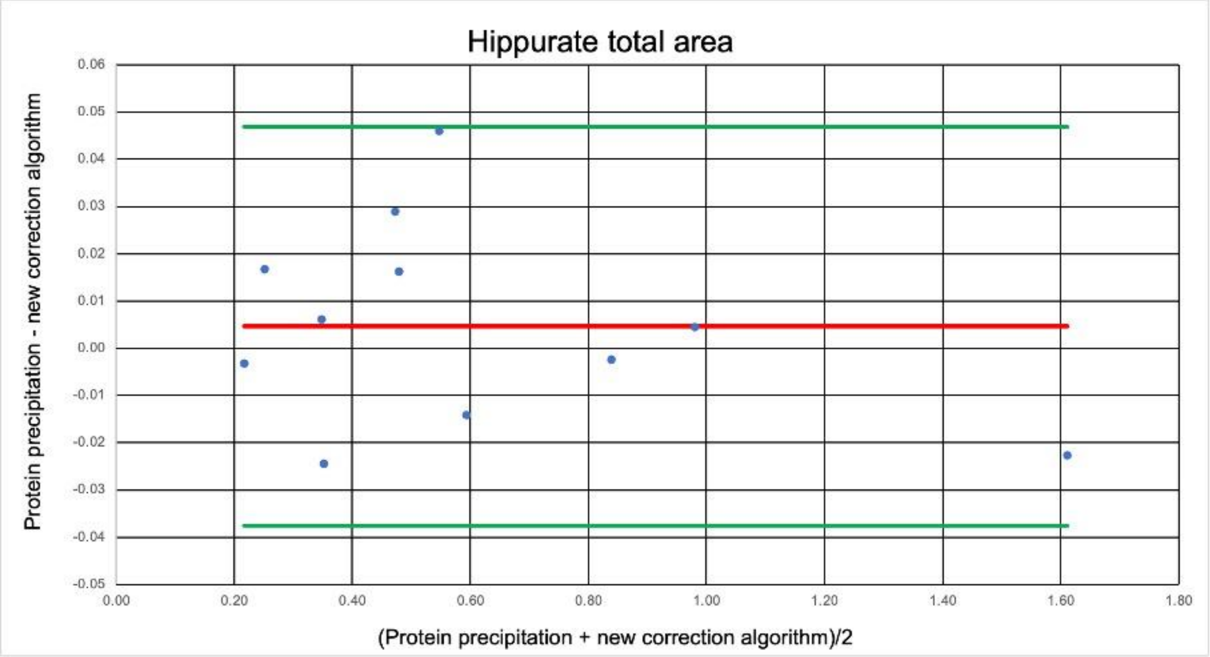

Figure S1. Comparison of the total area of 3-indoxyl sulfate (A) and hippurate (B) in urine specimens determined by the linewidth-based correction algorithm versus protein precipitation. Related to Figure 2.

The Bland-Altman plots show the differences between the peak integrals for total (A) 3-indoxyl sulfate and (B) hippurate determined by 1D ^{1}H CPMG NMR after protein precipitation of urine with methanol and the use of linewidth-based correction in native proteinaceous urine, respectively, in relation to the mean of the two measurements. The mean deviation between the two methods is indicated by the red line and the 95% confidence interval is indicated by the two green lines.

A

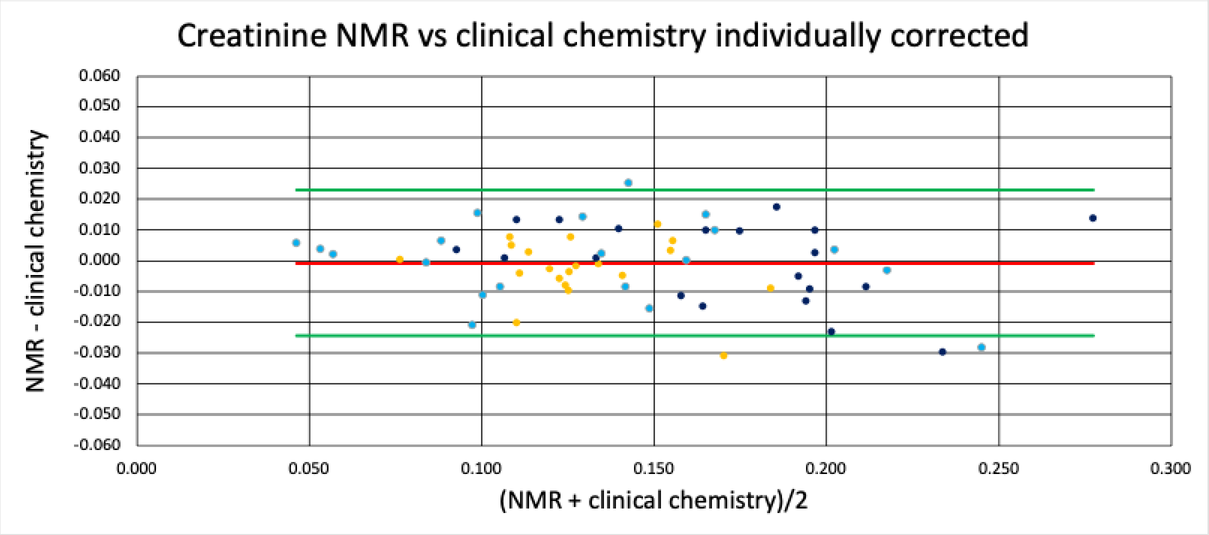

B

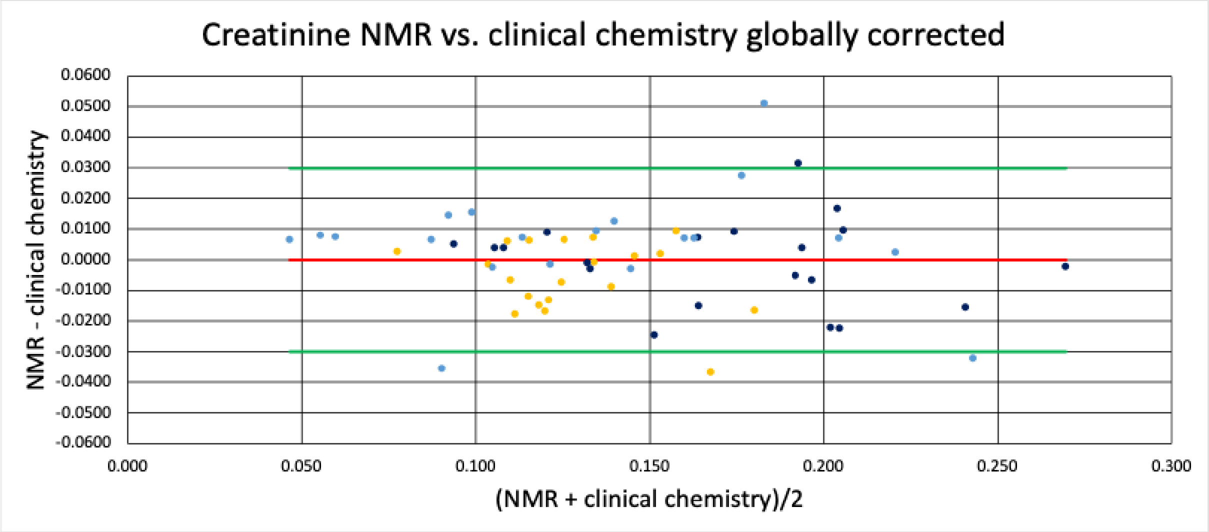

Figure S2. Differences between total plasma creatinine values obtained by NMR with (A) the application of individual linewidth-based correction factors and (B) the application of one global average linewidth-based correction factor with values from clinical chemistry. **Related to Table 1 and 2 and Figure 2.**

The mean deviation between the two methods is indicated by the red line and the 95% confidence interval by the two green lines. Specimens originating from patients with a UACR below 1.27, between 100 and 102, and above 6297 mg/g (albumin/creatinine) are color coded in orange, dark blue, and light blue, respectively.

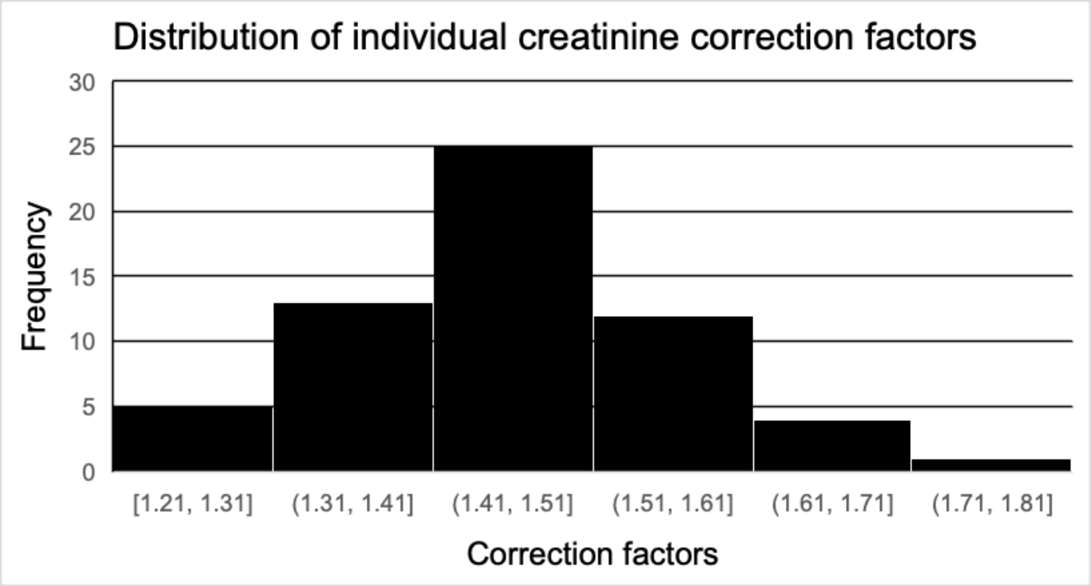

Figure S3. Distribution of individual correction factors for creatinine obtained for 60 plasma samples of the GCKD cohort. Related to Table 1 and 2 and Figure 2.

Correction factors range from 1.21 to 1.79 with a median of 1.46.

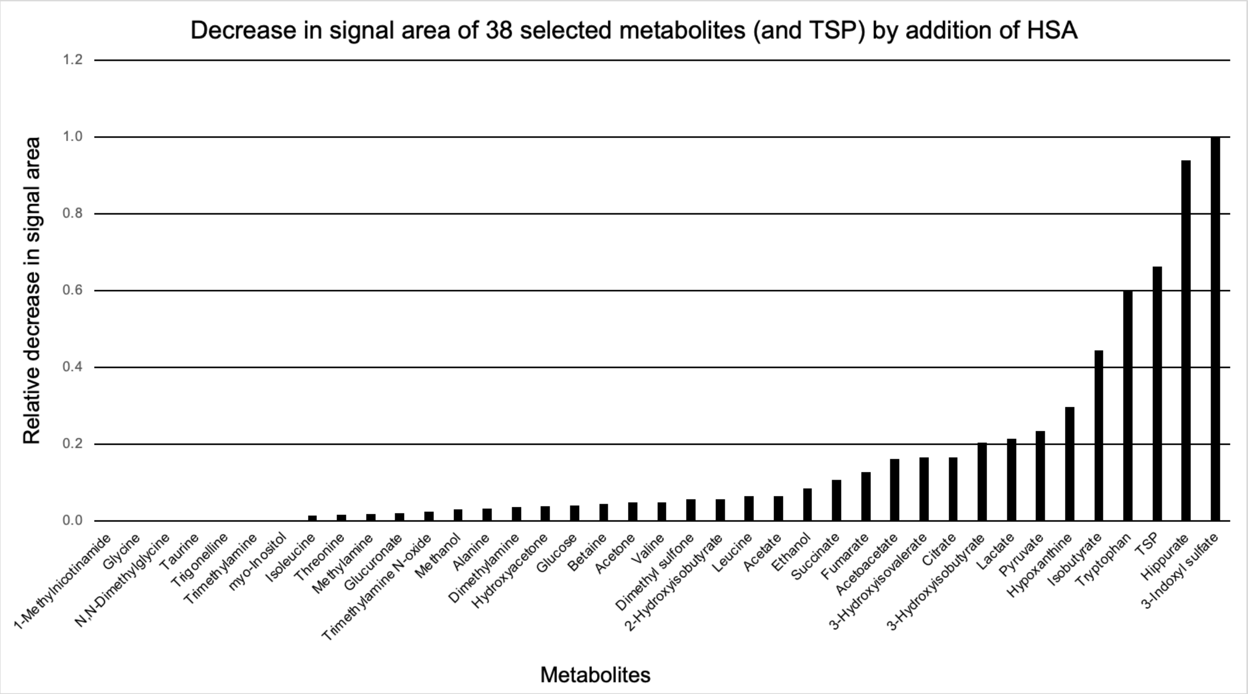

Figure S4. Differences in binding to albumin of 38 selected metabolites in a synthetic urinary matrix. Related to Figure 1.

The relative decrease in signal area in the presence of 18 g/L (0.27 mM) human serum albumin (HSA) is shown in ascending order, from the smallest to the largest decrease, for 38 metabolites and TSP. A value of one corresponds to a complete loss of signal, which reflects the complete binding of a metabolite to albumin.
